# Comparison of proteomic profiles in the zebrafish retina during experimental degeneration and regeneration

**DOI:** 10.1038/srep44601

**Published:** 2017-03-16

**Authors:** Karen Eastlake, Wendy E. Heywood, Dhani Tracey-White, Erika Aquino, Emily Bliss, Gerardo R. Vasta, Kevin Mills, Peng T. Khaw, Mariya Moosajee, G. Astrid Limb

**Affiliations:** 1 National Institute for Health Research (NIHR) Biomedical Research Centre at Moorfields Eye Hospital NHS Foundation Trust and UCL Institute of Ophthalmology, London, EC1V 9EL, United Kingdom; 2 Centre for Translational Omics, UCL Great Ormond Street Institute of Child Health, 30 Guilford St, London WC1N 1EH, United Kingdom; 3 Department of Microbiology and Immunology, University of Maryland School of Medicine and IMET, Columbus Center, 701 E, Pratt Street, 3061/3062, Baltimore, USA

## Abstract

Zebrafish spontaneously regenerate the retina after injury. Although the gene expression profile has been extensively studied in this species during regeneration, this does not reflect protein function. To further understand the regenerative process in the zebrafish, we compared the proteomic profile of the retina during injury and upon regeneration. Using two-dimensional difference gel electrophoresis (2D-DIGE) and label-free quantitative proteomics (quadrupole time of flight LC-MS/MS), we analysed the retina of adult longfin wildtype zebrafish at 0, 3 and 18 days after Ouabain injection. Gene ontology analysis indicates reduced metabolic processing, and increase in fibrin clot formation, with significant upregulation of fibrinogen gamma polypeptide, apolipoproteins A-Ib and A-II, galectin-1, and vitellogenin-6 during degeneration when compared to normal retina. In addition, cytoskeleton and membrane transport proteins were considerably altered during regeneration, with the highest fold upregulation observed for tubulin beta 2 A, histone H2B and brain type fatty acid binding protein. Key proteins identified in this study may play an important role in the regeneration of the zebrafish retina and investigations on the potential regulation of these proteins may lead to the design of protocols to promote endogenous regeneration of the mammalian retina following retinal degenerative disease.

Fish and amphibians have the ability to completely regenerate a functional retina after injury and this capability continues throughout adult life^1^,^2^. Although small mammals have a limited capacity for regeneration in early postnatal life^3^,^4^,^5^, there is no evidence of endogenous retinal regeneration occurring in humans. In contrast, upon injury, the human neural retina degenerates and undergoes gliosis and scar formation^6^. Elucidation of the complex cellular and molecular mechanisms that drive regeneration in the zebrafish may lead to identification of molecules that may be potentially targeted to promote endogenous regeneration of the human retina, avoiding cell transplantation procedures.

The zebrafish has been increasingly used as a model to study complex tissue regeneration of the retina. Several models of retinal insult in this species including surgical removal, light damage and neurotoxin damage result in complete recovery of the functional retina^7^,^8^,^9^. Neuronal regeneration is performed by resident stem cells known as Müller glia, and this process involves cell proliferation, migration to the lesioned site and differentiation to new neural retinal cells to restore the damaged area^10^. Since Müller glia with stem cell characteristics have been identified in the adult human retina and can be induced to grow and differentiate into retinal neurons *in vitro*^11^,^12^,^13^, there is potential for inducing re-activation of these quiescent cells *in vivo*. Currently, most of our understanding of the retinal regenerative process in the zebrafish is based upon gene expression of isolated retina^9^,^14^,^15^, which have identified many molecular mechanisms involved in this process. However, it is also important to explore the protein expression profile of the retina in this species as proteins determine the functional activity of the cell. Therefore protein studies would enhance our understanding of the active and functional mechanisms occurring during regeneration which may be potentially targeted in the human retina to promote endogenous repair.

The aim of the current study was to identify protein expression changes in the zebrafish retina that may regulate the regenerative processes after injury in this species. We compared the protein expression profile of normal zebrafish retina with that of retina damaged by intravitreal injection of ouabain undergoing degeneration and during the process of regeneration. We conducted 2D Difference Gel Electrophoresis (2D-DIGE) and label free proteomic analysis on these samples. We have shown that metabolic processing is negatively regulated, and fibrin clot formation was upregulated during retinal degeneration in the zebrafish. In addition, apolipoproteins A-Ib and A-II and the carbohydrate-binding protein extracellular matrix factor galectin-1 were highly upregulated in the degenerated zebrafish retina as compared to normal retina. Similarly, in the regenerating retina we observed activation of cytoskeletal and extracellular matrix proteins during regeneration. More specifically, cytoskeletal proteins such as tubulin beta 2A are highly upregulated in the regenerating zebrafish retina as compared to control. The validation of galectin-1 expression was supported by RT-PCR and western blot.

Factors identified are likely to be important for the regenerative process in the zebrafish retina and further investigations on the roles of these molecules in the mammalian retina may aid to design regenerative approaches to induce endogenous repair of the human retina.

## Results

### Validation of zebrafish retinal degenerative and regenerative stages after Ouabain injection

**Figure 1:**
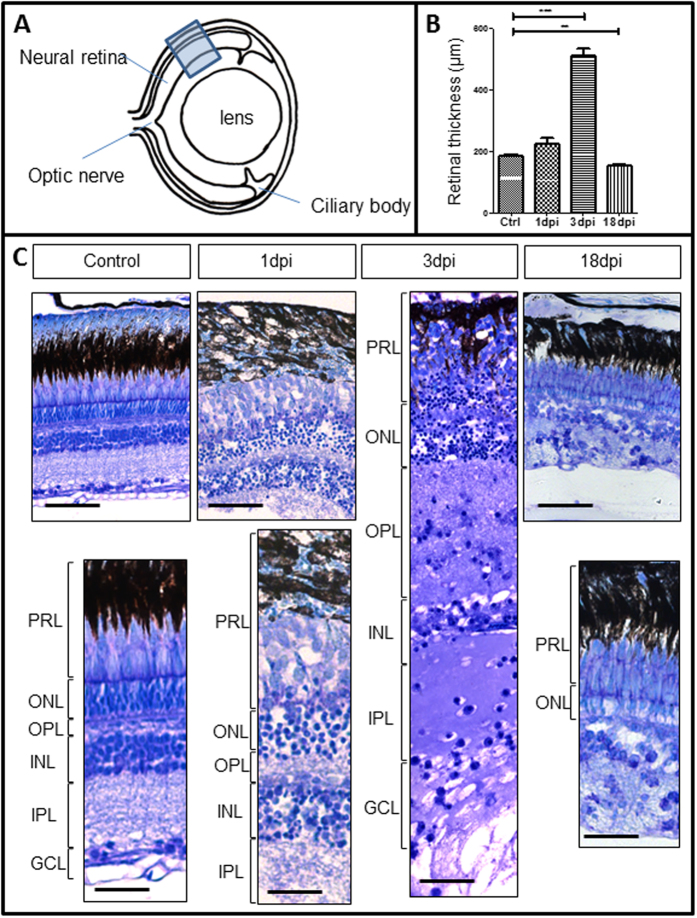
Comparison of zebrafish retinal structure between normal and Oubain damaged retina at 1, 3 and 18 days post injection (dpi). (**A**) Site of retinal sampling from which images in (**C**) were taken for consistency. (**B**) Histogram shows the differences in the mean neural layer thickness between control, 1 dpi, 3 dpi and 18 dpi (**P = 0.0044; ***P < 0.0001) n = 3. (**C**) Retinal sections were stained with toluidine blue. While the retina structure at 1 dpi appears less dense in comparison to control, the retina at 3 dpi almost doubled in size and cellularity was clearly decreased. By 18 dpi the photoreceptor layer had regained structure but the inner retinal layers could not be determined. PRL = photoreceptor layer; ONL = outer nuclear layer; OPL = outer plexiform layer; INL = inner nuclear layer; IPL = inner plexiform layer; GCL = ganglion cell layer. Scale = 50 μm.

Examination of the zebrafish retina after injection of 200 μM ouabain showed evident morphological changes occurring after toxin-induced damage. Bright field images were taken from the same site of the peripheral retina of treated and control animals at various time intervals after injection (Fig. 1A). The thickness of the neural retina was measured between control, 1 dpi, 3 dpi and 18 dpi. A highly significant increase in retinal thickness with decreased cell density was observed when comparing the control and 3 dpi (P < 0.0001). In contrast, a significant decrease in retinal thickness was observed between control and 18 dpi (P = 0.004) (Fig. 1B,C). At 1 dpi cells within the different retinal layers appeared dispersed, and cell nuclei were more diffuse and rounded. At 3 dpi a marked decrease in cell density was seen in all the three retinal cell layers, accompanied by a 2.8 times increase (p < 0.0001) in the neural retina thickness (Fig. 1B,C). This was thought to be the time at which the retina reached its maximum damage prior to the onset of regeneration. At 18 dpi a partial recovery of retinal morphology could be observed however its thickness is significantly reduced in size as compared with normal retina (p = 0.0044) (Fig. 1B,C). A photoreceptor layer could be seen at 18 dpi, although the inner retinal layers (INL, IPL and RGC) could not be distinguished, indicating that the retina was actively regenerating (Fig. 1C). Based on these observations, retinae obtained at 3 and 18 dpi were chosen for proteomic examination of the degenerated and regenerating retina respectively.

### Protein expression changes observed in the degenerated and regenerating zebrafish retina after retinal damage induced by ouabain

**Figure 2:**
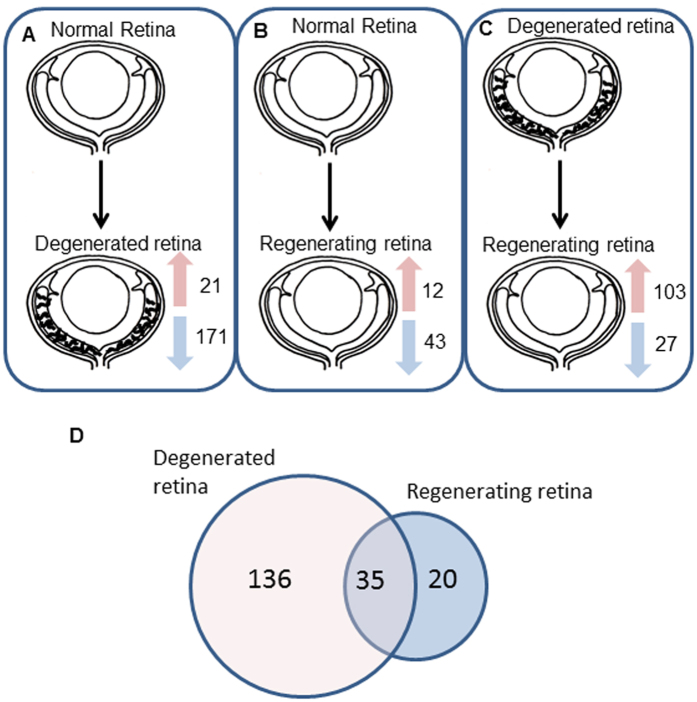
Differential expression of proteins between normal, degenerated and regenerating zebrafish retina. Numbers of proteins differentially expressed between (**A**) normal and degenerated retina, (**B**) normal and regenerating retina and (**C**) degenerated and regenerating retina. Red arrows indicate the number of upregulated proteins and blue arrows indicate the number of downregulated proteins that were differentially expressed. (**D**) Venn diagram shows the number of proteins differentially expressed in the degenerated retina and regenerating retina as compared to normal retina. Numbers in both circles are common to both degenerated and regenerating retina.

Analysis of the proteomic profile of zebrafish retina by label free mass spectrometry identified the presence of 2042 proteins in the control retina, 1514 proteins in the degenerated retina and 1445 proteins in the regenerating retina. Further examination of the proteins detected in the zebrafish retinae identified several proteins that were differentially expressed between control, degenerated and regenerating retina. Using the normalised abundancies, proteins considered as differentially expressed were either ≥2-fold upregulated or ≤0.5-fold downregulated between the different time points. In the degenerated retina, 192 proteins were found to be differentially expressed as compared to the control retina, with 21 being ≥2-fold upregulated and 171 being ≤0.5-fold downregulated (Fig. 2A). Fewer changes in expression were detected between control and regenerating retina, where 12 proteins were upregulated ≥2-fold and 43 downregulated ≤0.5-fold (Fig. 2B). Between the regenerating and degenerating retina, 130 proteins were also found to be differentially expressed, with 103 ≥ 2-fold upregulated and 27 ≤ 0.5-fold downregulated in the regenerating retina (Fig. 2C). Of the proteins found to be differentially expressed in the degenerated or regenerating retina as compared to control retina, 35 were common in both specimens (Fig. 2D).

### Identification of individual proteins differentially expressed in the degenerated and regenerating retina reveals major changes in cytoskeletal proteins, histones and apolipoproteins

Individual protein analysis showed that only one protein, annexin A2A, was ≥2-fold upregulated in both the degenerated and regenerating zebrafish retina as compared to control. In the degenerated retina the glycoprotein vitellogenin 6 exhibited the highest fold upregulation when compared to control retina (32.12-fold increase) (Table 1). This was followed by the signalling molecule fibrinogen gamma polypeptide (13.05-fold) and vitellogenin 1 (8.60-fold increase). Many proteins differentially expressed in both degenerated and regenerating retina were cytoskeletal related proteins. In the regenerating retina, the cytoskeletal protein tubulin beta 2A class IIa exhibited the highest fold increased expression (44.24-fold) as compared to control. This was followed by brain type fatty acid binding protein (4.82-fold) and histone H2B (4.41-fold). Another intermediate filament protein, vimentin, which showed a 0.35-fold downregulation in the degenerated zebrafish retina, was found 2.59-fold upregulated in the regenerating zebrafish retina as compared to control. Proteins involved in nucleic acid binding were also highly represented and included ubiquitin A 52 residue ribosomal protein fusion product 1 and two forms of histone H2B (Table 1). Three different isoforms of histone H2B (Accession: G1K2L0, Q6DH91, F1QIH4), were found to be expressed at different levels. It was also observed that Q6DH91 and F1QIH4 were ≥2-fold upregulated in the regenerating retina as compared to control retina, whilst there was no difference in the expression between control and degenerated retina. Histone H2B isoform G1K2L0, however showed ≥2-fold upregulation in the degenerated retina as compared to control retina, but no difference was observed between regenerating and control retina (Table 1). Supplementary Table 1 contains a complete list derived from label –free mass spectrometry analysis as presented by Progenesis software.

### Differential protein expression in control, degenerated and regenerating zebrafish retinae as identified by 2D-DIGE analysis

**Figure 3:**
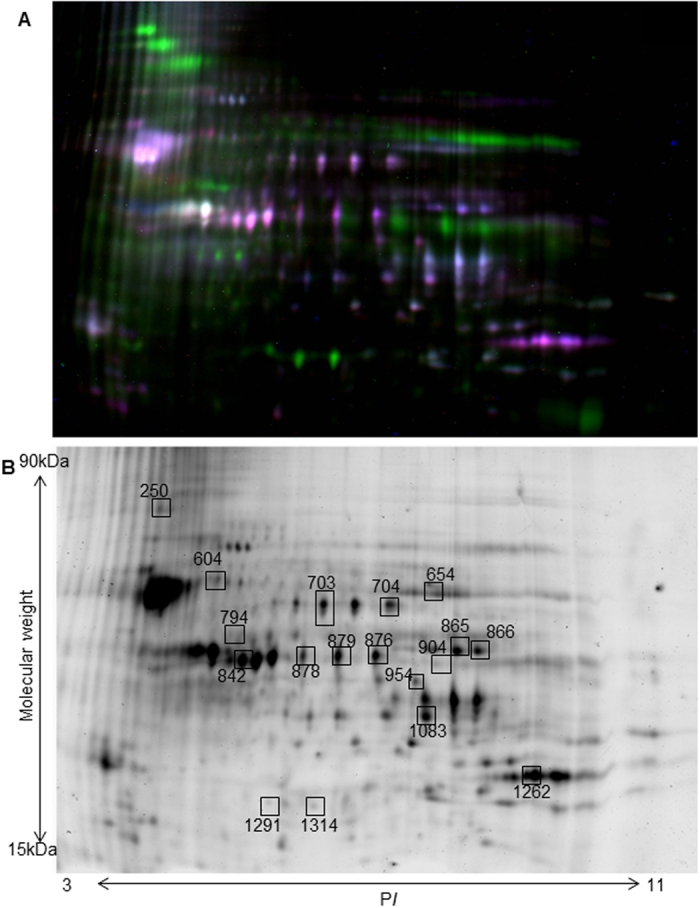
2D gel DIGE analysis of normal, degenerated and regenerating zebrafish retina. (**A**) Fluorescent gel image of protein spots from zebrafish retina. Normal zebrafish retina (Cy3/Red), degenerated retina (Cy5/Blue) and regenerating zebrafish retina (Cy2/green) are represented in the gel. (**B**) Representative image of protein spots from normal zebrafish retina. Numbered spots indicate those which were identified to be differentially expressed.

Approximately 135 spots were detected in the 2D-DIGE gel containing samples of control, degenerated and regenerating zebrafish retinae (Fig. 3). Comparison between the spot intensities of the three groups of proteins identified 67 spots which were differentially expressed. In the degenerated retina 24 spots showed a ≥2-fold upregulation, whilst 36 showed a ≤0.5-fold downregulation as compared to control retina. In the regenerating retina, 38 proteins showed a ≥2-fold upregulation and 24 showed ≤0.5-fold downregulation as compared to the degenerated retina. No spots were found to be differentially expressed between control and regenerating retina. Spots with the highest intensity changes were extracted (18 selected spots) for further analysis by mass spectrometry and protein identification (Fig. 3). Those proteins found upregulated in the degenerated retina as compared to control and regenerating retina were identified as apolipoprotein A-Ib, fibrinogen beta polypeptide, and heat shock protein 90-alpha 2. Several proteins were also identified to be downregulated in the degenerated retina as compared to control and regenerating retina and included fructose bi-phosphate aldolase, internexin neuronal intermediate filament protein, enolase 1, ES1 protein, plectin a, arrestin 3a, aspartate aminotransferase and brain subtype creatine kinase (Table 2).

### Gene ontology and pathway enrichment indicates a reduction in energy homeostasis and increased fibrin clot formation during retinal damage

**Figure 4:**
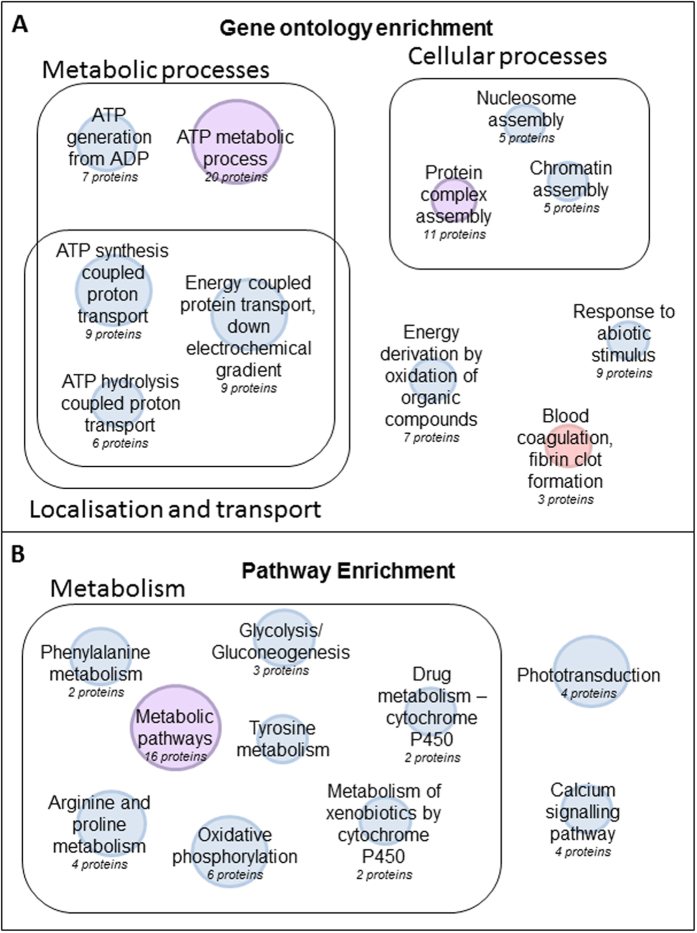
Gene ontology and pathways significantly enriched in the degenerated zebrafish retina as compared to normal retina. Representation of (**A**) gene ontology terms and (**B**) KEGG pathways which were significantly enriched in the degenerated zebrafish retina as determined by Webgestalt and Panther databases. The size of the node represents the significance of enrichment, where the larger the node the higher the significance. The node colour represents whether the proteins within this group are >2-fold upregulated (red), <0.5-fold downregulated (blue) or contain both up and downregulated proteins (purple).

Proteins that were differentially expressed between degenerated and control zebrafish retina by 2D-DIGE and label free proteomics were subject to gene ontology (GO) enrichment and pathway analysis to identify key functional areas of interest. Figure 4 shows a diagram illustrating significantly enriched gene ontology groups for this dataset. Due to the zebrafish databases being incomplete, 54 protein IDs could not be mapped to the ontology databases. Despite this, analysis revealed an over-representation of proteins grouped to ATP metabolic processes, blood coagulation, nucleosome assembly, protein assembly and response to abiotic stimulus (Fig. 4A). Proteins involved in ATP metabolic processes included 20 proteins to which all but 1 were ≤0.5-fold downregulated in the degenerated retina as compared to control retina. The only protein which was ≥2-fold upregulated in this group was identified to be an adenylate kinase isoenzyme (Q68EH2). Proteins involved in blood coagulation and fibrin clot formation included fibrinogen gamma polypeptide, fibrinogen beta polypeptide and fibrinogen alpha chain, and were observed ≥2-fold upregulated in the degenerated retina as compared to control retina. Of the nine proteins involved in response to abiotic (non-living) stimulus, all were downregulated in the degenerated retina and included retinal proteins recoverin, cone transducin subunit alpha, and the heat shock protein 90 alpha-2. Pathway enrichment analysis of this protein group also showed that metabolic regulation was highly represented. In total, 19 proteins were associated to various metabolic regulatory pathways, which were observed negatively regulated. Other pathways enriched included photo-transduction and the calcium signalling pathway. Interestingly, all proteins involved in these pathway groups were identified to be ≤0.5-fold downregulated in the degenerated zebrafish retina with the exception of adenylate kinase, found in metabolic pathways, which was ≥2-fold upregulated (Fig. 4B). Details of p-values and FDR are provided in Supplementary Figure 2.

### Dynamic changes in GTPase activity, cytoskeletal regulation and membrane transport during retinal regeneration as shown by gene ontology and pathway enrichment

**Figure 5:**
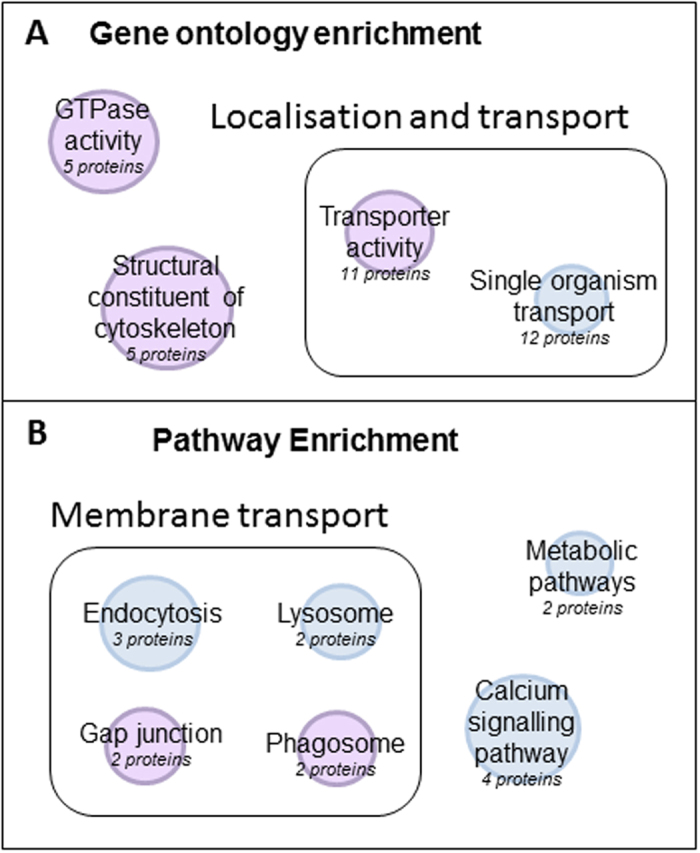
Gene ontology and pathways significantly enriched in the regenerating zebrafish retina as compared to normal retina. Representation of (**A**) gene ontology terms and (**B**) KEGG pathways which were significantly enriched in the regenerating zebrafish retina as determined by Webgestalt and Panther databases. The size of the node represents the significance of enrichment, where the larger the node the higher the significance. The node colour represents whether the proteins within this group are <0.5-fold downregulated (blue) or contain both up and downregulated proteins (purple).

Differentially expressed proteins between regenerating retina (18 dpi) and control retina obtained from the label free mass spectrometry were also subject to gene ontology and pathway enrichment. Out of the 55 proteins in this dataset, 16 could not be mapped by the enrichment. It was shown that proteins involved in the structural constituent of the cytoskeleton were the most significantly enriched within this group. These proteins included tubulin alpha 1B chain and an uncharacterised tubulin (Q6PE34), which were ≤0.5-fold downregulated and tubulin beta 2B chain, tubulin beta 5, and vimentin, which were ≥2-fold upregulated in the regenerating retina as compared to control retina. Proteins involved in GTPase activity and transporter activity were also significantly enriched (Fig. 5A). Interestingly pathway enrichment analysis showed that proteins involved in membrane transport were highly represented. In total, all proteins apart from tubulin beta 5, found in both gap junction and phagosome pathways were ≤0.5-fold downregulated in the regenerating retina as compared to control retina (Fig. 5B). Details of p-values and FDR are provided in Supplementary Figure 3.

### Validation of the proteomic results by PCR and western blot

**Figure 6:**
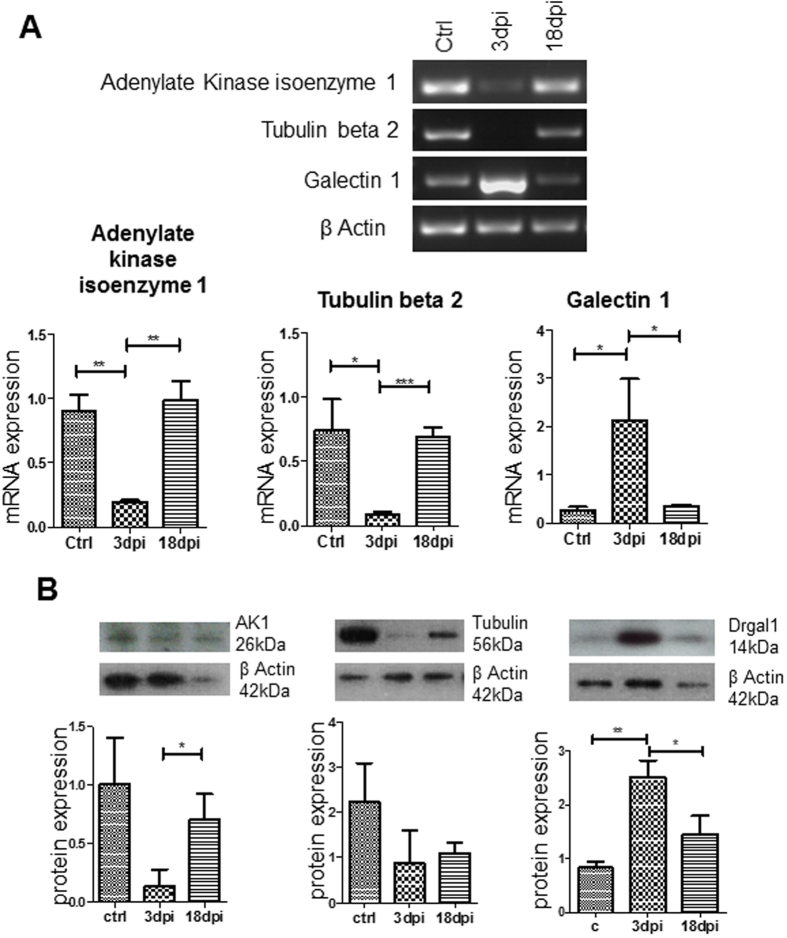
PCR and western blot validation of results generated by proteomic analysis of degenerated and regenerating zebrafish retina. (**A**) Shows representative PCR gel bands and histograms for adenylate kinase, tubulin beta 2, galectin 1 normalised to housekeeping gene beta actin for control, degenerated (3 dpi) and regenerating (18 dpi) retina (n = 3) (***P < 0.001; **P < 0.005; *P < 0.05). (**B**) Shows representative western blot protein bands and histograms for adenylate kinase, tubulin beta 2 and galectin 1 normalised to beta actin for control, degenerated and regenerating retina (n = 3) (**P < 0.005; *P < 0.05).

To validate our MS proteomic results, we conducted PCR and western blot analyses to examine the mRNA and protein expression. We selected three of the highly upregulated proteins to which antibodies were readily available for the zebrafish, including adenylate kinase isoenzyme1, tubulin beta 2 and galectin 1. Analysis of mRNA for galectin 1 correlated with proteomic results, and showed a significant increase in expression in the degenerated retina (3 dpi) as compared to normal retina (P = 0.0461). In contrast, mRNA levels of tubulin beta 2 did not correlate to the proteomics results and showed a significant decrease in the degenerated retina as compared to control (P = 0.0270) that returned to normal levels in the regenerating retina. A significant reduction in mRNA expression of adenylate kinase isoenzyme was observed in the degenerated retina as compared to control, and regenerating retina (P < 0.0020) (Fig. 6A). Similarly, protein expression of galectin 1 confirmed mass spectrometry results and showed a significant upregulation in the degenerated retina as compared to control (P < 0.0033) and regenerating retina (P < 0.0401). Protein expression of tubulin appeared to decrease in the degenerated retina as compared to control but not at a significant level. A significant reduction in the protein expression of adenylate kinase was observed in the degenerated retina as compared to regenerating retina (P < 0.0480) (Fig. 6B).

## Discussion

This study has applied a proteomics approach to investigate molecular events occurring during degeneration and regeneration in the zebrafish retina. We observed that upon degeneration proteins involved in fibrin deposition and cytoskeletal proteins were highly upregulated, whereas energy metabolism in the retina is mostly negatively regulated. Similarly in the regenerating retina, cytoskeletal proteins such as tubulin beta 2 were highly upregulated and proteins involved in transporter activity were downregulated. Validations of galectin1 by western blot and RT-PCR correlated with results. However mRNA and protein expression of tubulin beta 2 and adenylate kinase largely conflicted with proteomic findings. The differences for beta tubulin 2 expression can be accounted for as fragments of this particular protein was identified across seven fractions and then averaged, as generated by Progenesis software. In comparison, galectin1 and adenylate kinase was identified in only one out of the ten fractions analysed. It is also well documented that mRNA and protein expression levels may differ in the same system^16^, and this highlights the importance to study both aspects of cellular functions. It is also worth noting that post translational modifications (PTMs) greatly affect protein activity^17^ and these may play a role in the regenerative response in the zebrafish retina, as shown in previous studies^18^. It would be therefore important to explore PTMs in future investigations. Overall, results from our study suggest that a dynamic protein expression pattern is required for the regenerative response and it would be beneficial to explore the role of these factors in mammalian retina to investigate their potential for promoting endogenous regeneration in these species.

Zebrafish retinae are small in size (1 mm × 1 mm), hence samples used in this study were pooled to obtain a single representative sample. Creating a sample pool can also reduce the sample bias and biological variance, leading to more precise data estimates^19^. The zebrafish proteome database presented in Uniprot is far from complete, and therefore limited the complete identification of all proteins in the given samples. Despite these limitations, this study identified protein expression changes in the zebrafish retina that may play key roles in the regenerative process in this species.

Analyses of zebrafish retina by 2D-DIGE and label-free mass spectrometry were comparable, supporting the validity of the results. Data presented by 2D-DIGE analysis showed that apolipoprotein A-1b (ApoA1b), fibrinogen beta polypeptide, and heat shock protein 90-alpha2 were ≥2-fold upregulated in the degenerating retina as compared to the control retina. These results supported the findings from the label free mass spectrometry analysis, which also showed upregulation of apolipoproteins ApoA2, ApoA1b and ApoC in the degenerated retina. Previous observations have also shown increased apolipoprotein expression, including ApoA1 and ApoE, after injury in zebrafish tissues, that are capable of regeneration including the eye and fin^20^,^21^. Apolipoproteins bind to lipids and regulate their metabolism and transport, and it is possible that these may play an important role in lipid re-organisation to support the regenerative process.

Gene ontology and pathway enrichment analyses of zebrafish proteins can be challenging due to the incomplete proteome database for this species. This may lead to partial or incorrect annotation of the dataset, and results should therefore be carefully analysed. Despite these limitations gene ontology analysis showed that proteins involved in energy metabolism were highly represented. However, in both the degenerated (3 dpi) and regenerating (18 dpi) retina the majority of these proteins were negatively regulated in comparison to control retina. Metabolic regulation in the retina is tightly controlled and is one of the most energy demanding tissues, with photo-transduction, neurotransmitter signalling, oxygen consumption and glucose metabolism all playing a significant role in energy consumption^22^. Although reduction in metabolic processes prior to the onset of retinal degeneration has previously been reported in the drosophila^23^, the role of these processes during regeneration of the retina has not been examined. Metabolism does however, play an important role in the regulation of stem cell proliferation and differentiation. During development in the embryonic xenopus, differentiating retinal progenitor cells rely on oxidative phosphorylation rather than glycolysis for energy production^24^. In addition, adult neural stem cells in mammalian tissues are known to utilise oxidative metabolism rather than glycolysis for cell fate specification^25^,^26^. Studies on stem-cell like Müller glia, which are responsible for retinal regeneration, have shown that they are resistant to hypoxia and that their energy metabolism is reliant on anaerobic glycolysis^27^, which might be linked to their stem cell activity. Interestingly, the present observations showed that among the metabolic proteins identified, adenylate kinase isoenzyme 1 was the only upregulated protein in the degenerated retina. This isoenzyme plays a key role in the metabolic monitoring of the cell, generating AMP signals from the conversion of ATP to ADP, where AMP acts as a metabolic signal to regulate energy production^28^, and may be important in the regulation of the regenerative response. The dynamic state of energy metabolism as shown in this study may therefore reflect the changing needs of stem cells driving proliferation and differentiation, a requirement for regeneration. Modification of metabolic pathways may therefore constitute important targets when investigating approaches to promote endogenous regeneration.

Vitellogenin 6, a glycoprotein involved in the response to activation of oestrogen receptors, showed the highest fold upregulation in the degenerated retina as compared to control retina. Although vitellogenin has been mainly described in the embryo and early developmental stages, expression has also been observed in adult tissues such as skin, brain and eye, however the function in these tissues is largely unknown^29^,^30^. This glycoprotein has been characterized as an aging marker in honey bees^31^, as well as a biomarker for estrogenicity in Platichthys flesus^32^. In addition, ovariectomized grasshoppers have shown higher expression levels of vitellogenin protein that correlates with long life due to reduced reproduction^33^. Interestingly, *in vitro* investigations of vitellogenins have shown hemagglutination activity, as well as inhibition of bacterial growth^34^, which may suggest a role in immune functions. However, from the present data, it is not possible to suggest a specific role for this glycoprotein during retina degeneration in the zebrafish, and this warrants investigations.

Many changes were observed in the regulation of cytoskeleton proteins between the control, degenerated and regenerating retina. Specifically, striking changes in the expression of several tubulins were observed between the control, degenerated and regenerating retina, with tubulin beta 2A showing the greatest fold increase in the regenerating retina as compared to control. Various tubulins are involved in retina remodelling and previous studies have shown changes in α-1 tubulin levels in Müller glia from the regenerating zebrafish retina^35^ that are thought to be important for the structural changes during regeneration. In addition beta tubulin levels have also found to be upregulated in rodents after axotomisation of RGC, which correlate to regenerative growth in neurons^36^. Interestingly, in our study the intermediate filament protein vimentin was observed downregulated in the degenerated retina, but upregulated during regeneration when compared to control. Vimentin is a type III intermediate filament protein expressed primarily by astrocytes and Müller glia in the retina. It plays a role in the organisation of organelles in the cytoplasm, provides resistance to mechanical stresses and maintain the cellular structure^37^. Upregulation of vimentin in Müller glia has been associated with cellular stress and is thought to contribute to the formation of the glial scar in the mammalian retina in response to disease or injury^37^,^38^. If the upregulation of vimentin is associated with glial cell proliferation, it could be suggested that high levels of vimentin may reflect the active Müller glia/progenitor proliferation that contribute to the formation of the new retinal structure in the regenerating zebrafish retina. Recent studies have highlighted the role that Müller glia plays in the injured zebrafish retina, where persistent gliosis diminishes the ability of these cells to induce regeneration^39^. In addition, initial upregulation of vimentin in the adult zebrafish brain after injury has been observed, suggesting that early responses to CNS lesions present characteristics of gliosis^40^. Although Müller glia proliferation and gliosis is observed after injury or disease in the human retina, these do not lead to regeneration. Therefore, understanding the mechanisms that control changes in the expression of intermediate filaments such as vimentin and tubulin could provide an insight into the remodelling of the retina following injury.

Neural retina remodelling may also occur through extracellular matrix regulation. Galectin 1, a beta galactoside-binding protein, was observed to be 2-fold upregulated in the degenerated zebrafish retina as compared to controls. This protein is expressed in the cytoplasm and can be translocated into the nucleus, or secreted into the extracellular space, where it can bind to cell surface. Galectin-1 plays a role in cell adhesion, transmembrane signalling and cell-cell interactions as well as inflammatory responses^41^. Galectin 1 has been specifically found in microglia and proliferating Müller glia after photoreceptor cell death in the zebrafish retina, and shown to participate in rod regeneration^42^. It has been hypothesised that this protein, which has the ability to inhibit vitronectin and chondroitin sulphate binding to the ECM, may play an important role in the repair of the zebrafish retina by providing a permissible environment for regeneration.

In conclusion, comparison of protein expression profile between the degenerated and regenerating zebrafish retina has highlighted key differences in protein expression. The data presented suggest that retinal regeneration in this species is likely to involve complex cellular processes involving proliferation, remodelling and metabolic changes, which might influence one another throughout the process. The study has highlighted proteins that may be important to examine for their function in order to create a permissible environment for endogenous regeneration of the mammalian retina.

## Methods

### *In vivo* intravitreal injection of Ouabain into the zebrafish eye

#### Injection protocol

Longfin wildtype Zebrafish, 7–9 months were obtained from UCL biological services Fish facility and maintained according to local and Home Office regulations for the care and use of laboratory animals and the UK (Scientific Procedures) Act at the UCL Institute of Ophthalmology. UCL approved all procedures for experimental protocols, which were conducted in accordance with the approved guidelines at UCL Institute of Ophthalmology. Zebrafish were anaesthetised using 1 mg/ml Tricane in aquarium water for 1 min. Injections were performed by making an incision into the temporal side of the zebrafish eye using a double edge sapphire blade (0.75 mm; WPI, UK), where the needle was inserted and Ouabain (Sigma Aldrich, UK) was injected intravitreally. The needle set up consisted of a 10 μl Gastight Hamilton syringe (Hamilton, Switzerland) with a retinal pigment epithelium (RPE) kit (WPI, UK). A total of 0.2–0.4 μl of 200 μM Ouabain in injectable saline solution was injected into each eye to produce a final intravitreal concentration of 10 μM. The protocol used was based upon the original paper by Sherpa *et al*., in which an injection of 200 μM was given to yield a final concentration of 10 μM in the vitreous^43^. To determine the optimum time point at which the zebrafish degenerate and regenerate in response to Ouabain toxicity, animals were culled at various times after injection and the retina examined histologically at 1, 3 and 18 days post injection (dpi). Having determined that at 3 dpi severe degeneration had occurred and that regeneration could be observed at 18 dpi, we pooled 30 retinae from each group to assess differences in the proteomic profile of the degenerated and regenerated retina as compared to control retina.

#### Retinal Histology

Zebrafish eyes were fixed for 24 hrs in 4% paraformaldehyde (PFA) at 4 °C. They were dehydrated through a graded ethanol series and then embedded in JB-4 plastic resin as described previously^44^. A Leica microtome was used to cut 12 μm sections, which were mounted on polytetrafluoroethene microscope slides. Sections were stained with 1% toluidine blue and sealed with DPX mountant and glass coverslips. Sectioned were imaged using a Leica DMRB with Jenoptik D-07739 Optical System. The mean thickness of the normal, degenerated and regenerating retina was measured under a light microscope and the average of three repeat measures in three retinal specimens were calculated for analysis.

### Proteomics analysis of zebrafish retina

#### Label Free proteomics

Retinal samples were washed in phosphate buffered saline (PBS) prior to homogenisation in the presence of 50 nM Ammonium Bicarbonate + 2%ASB-14 (Sigma Aldrich, UK) pH8.2, followed by sonication. Protein content was estimated using the Bicinchoninic Acid protein assay kit (Sigma-Aldrich, UK). Samples were pooled to obtain three separate conditions, consisting of control, degenerated and regenerating zebrafish retina to a total of 80 μg. Samples were run on a 1D gel (BioRad Mini Protean Precast gels, BioRad, UK) as previously described^45^. Ten gel bands of each sample lane were excised according to molecular weight and processed for protein digestion using trypsin (Sigma, UK) and peptides extracted using standardized methods^46^. Samples were reconstituted in 3% acetonitrile (ACN) + 0.1% trifluoroacetic acid (TFA) and 50 fmols Massprep yeast enolase (Waters, UK) was spiked into the protein preparation, acting as an internal standard. All analyses were performed as described previously^46^ using a QToF Premier™ mass spectrometer coupled to a NanoAquity Nano-LC system (Waters Corp, UK).

#### Data analysis

Data was processed using ProteinLynx Global Server (PLGS) 2.4 software (Waters, UK) and peptides were matched using downloaded Uniprot reference proteome for Danio rerio. The protein sequence for the internal standard, yeast enolase1 (Accession: P00924) was also added to the database. Identified peptide data was imported into Progensis LC-MS (non-linear dynamics) for comparative analysis. Peptides were normalised to housekeeping protein beta actin (E9QD59). Further bioinformatics analysis of the results were conducted on web-based platforms which included the Panther Classification System (http://www.pantherdb.org/), and gene ontology classification by WebGestalt (http://bioinfo.vanderbilt.edu/webgestalt/).

Samples from 30 eyes were pooled for each group to assess differentially expressed proteins between the three conditions studied (control, degenerated, and regenerating), given an n number of 1 for each condition. Therefore the high cut-off values of ≥2-fold upregulated or ≤0.5-fold downregulated were chosen to obtain higher confidence in analysing the data from a pooled cohort.

The mass spectrometry proteomics data have been deposited in the ProteomeXchange Consortium database (http://proteomecentral.proteomexchange.org) via the PRIDE partner repository^47^ with the dataset identifier PXD005843 and DOI 10.6019/PXD005843.

### 2D- differential gel electrophoresis (DIGE)

Isolated zebrafish retinal protein used for the mass spectral analysis was also used in the 2D DIGE analysis. As before, samples were pooled to create 3 separate conditions consisting of 150 μg protein for each the control, degenerated and regenerating retina. 2D DIGE analysis was performed as previously described^45^. Briefly, pooled samples were freeze dried overnight, reconstituted in DIGE buffer (7 M Urea, 2 M Thiourea, 2% CHAPS, 10 mM Tris-HCL pH 8.3), vortexed and centrifuged briefly. Samples were labelled with 600 pmol of each Cy 3 (control retina), Cy5 (degenerated retina) and Cy2 (regenerating retina). The reaction was halted by addition of 1 ul 10 mM lysine to each sample and the 3 Cy dye labelled samples combined. Combined samples were added to IPG strips in rehydration solution (7 M urea, 2 M thiourea, 2% CHAPS, 20 mM DTE, 20% resolyte + trace bromophenol blue) and run on an IPG Multiphor (GE Healthcare, Little Chalfont, U.K.) at a gradient (30 V 0.05 hr, 300 V 1 hr, 1000 V 1 hr, 8000 V 4 hr, 8000 V 24 hr hold). For the second dimension, 12% acrylamide gels were cast and IPG strips re-equilibrated. Strips were resolved on an Ettan DALT twelve System separation tank and run overnight at 2 watts per gel in Tris-glycine-SDS(25 mM–198 mM-01% w/v). Gels were fixed (50% methanol, 7% acetic acid) and imaged using a Typhoon scanner (Model 8600, GE Healthcare, UK). Spot comparison was performed using Progenesis Samespots software (Non-Linear Dynamics, Waters, UK). Gels were silver stained as described previously^45^ and protein spots excised and trypsin digested. Peptide extraction was then performed as described for label free proteomics above.

## Additional Information

**How to cite this article:** Eastlake, K. *et al*. Comparison of proteomic profiles in the zebrafish retina during experimental degeneration and regeneration. *Sci. Rep.* **7**, 44601; doi: 10.1038/srep44601 (2017).

**Publisher's note:** Springer Nature remains neutral with regard to jurisdictional claims in published maps and institutional affiliations.

## Supplementary Material

Supplementary Tables

## Figures and Tables

**Table 1:** Differentially expressed proteins between normal, degenerated and regenerating zebrafish retina as identified by mass spectrometry.

Protein Class	Accession	Protein name	Peptide count	Fold change		
				D/N	R/N	R/D
Calcium binding protein	Q6XG62	Ictacalcin	6	1.21	**3.90**	**3.22**
Cell adhesion molecule	E9QFY9	Galectin	3	**5.02**	1.64	*0.33*
Cytoskeletal	F1QKY8	Tubulin, beta 2A class IIa	91	0.56	**44.24**	**79.16**
	Q6NW90	Tubulin beta 5	138	0.51	**4.04**	7.85
	F1QCY2	Tubulin alpha 8 like 3	107	**3.47**	*0.13*	*0.037*
	F1QAM8	Vimentin	5	*0.35*	**2.59**	7.33
	F1RCB6	Actin	75	**2.27**	1.73	0.76
	Q5XTP0	Crystallin gamma S1	3	**4.21**	0.59	0.14
	Q52JI4	Beta B2 crystallin	10	**2.26**	0.60	*0.27*
	Q6P3K5	Krt5 protein	2	1.97	**2.01**	1.02
Kinase/ Transferase	Q68EH2	Adenylate kinase isoenzyme 1	2	**2.00**	0.54	*0.27*
	Q50LC6	Glutathione S transferase pi 2	14	**2.12**	0.73	*0.34*
Nucleic acid binding	F1Q912	Predicted: Transcription co-factor	2	**7.96**	0.71	**0.089**
	Q3B7P7	Ubiquitin A 52 residue ribosomal protein fusion product 1	54	1.32	**4.17**	**3.16**
DNA binding	G1K2L0	Histone H2B	52	**4.76**	0.76	*0.16*
	Q6DH91	Histone H2B	16	1.47	**4.41**	**3.00**
	F1QIH4	Histone H2B	67	1.78	**2.28**	1.28
Signalling molecule	Q7ZVG7	Fibrinogen gamma polypeptide	109	**13.05**	0.74	*0.056*
	Q6NYE1	Fibrinogen B beta polypeptide	90	**6.32**	0.99	*0.16*
	B8A5L6	Fibrinogen alpha chain	93	**6.70**	1.30	*0.19*
Glycolipoprotein	F1QV15	Vitellogenin 6	51	**32.12**	1.28	*0.04*
	F1R887	Vitellogenin 4	52	*0.09*	**2.50**	**28.83**
	Q1MTC4	Vitellogenin 2	6	**4.55**	1.07	*0.23*
	Q1LWN2	Vitellogenin 1	60	**8.60**	*0.32*	*0.04*
Apolipoprotein	E7FES0	Apolipoprotein A-Ib	32	**7.00**	0.77	*0.11*
	B3DFP9	Apolipoprotein A-II	16	**6.11**	0.84	*0.14*
	E9QDI1	Apolipoprotein C-I like	12	**3.88**	0.73	*0.19*
Transport proteins	B8JL43	Serotransferrin	8	**5.58**	1.23	*0.22*
	Q66I80	Fatty acid binding protein 11a	27	**2.25**	0.78	0.35
	I3ISI4	Predicted: fatty acid binding protein 3	10	0.80	**2.81**	**3.53**
	Q9I8N9	Brain type fatty acid binding protein	36	0.66	**4.82**	**7.35**
	Q6P603	Annexin A2A	12	**6.70**	**3.34**	*0.50*

Table shows proteins which exhibited 2-fold increase in expression in the degenerating as compared to normal retina as well as those in the regenerating over normal and degenerated retina.

(Numbers in Bold = 2-fold or more upregulated; Numbers in *italics* = 0.5-fold or less downregulated). N = normal retina; D = degenerated retina; R = regenerating retina.

**Table 2:** Differentially expressed proteins between normal, degenerating and regenerating zebrafish retina as identified by 2D-DIGE.

Spot No.	Accession	Peptide count	Protein Name	Fold change		
				R/D	D/N	R/N
954	B2GP30	17	Fructose-bisphosphate aldolase	**2.18**	*0.51*	1.11
604	F1Q8F1	10	Internexin neuronal intermediate filament protein	**2.45**	*0.67*	1.64
865	Q7ZUW8	10	Aspartate aminotransferase	**2.65**	*0.29*	0.71
866	Q6PC12	5	Enolase 1, (alpha)	**2.12**	*0.34*	0.72
1262	Q0D274	16	ES1 protein, mitochondrial	**4.28**	*0.27*	1.14
1083	E7F8G7	5	Plectin a	**2.21**	*0.41*	0.91
842	Q8AY63	28	Brain-subtype creatine kinase	**3.12**	*0.43*	1.35
879	Q6DH07	23	Arrestin 3, retinal (X-arrestin), like	**2.20**	*0.46*	1.00
703	Q6IQP5	36	Enolase 1, (Alpha)	**2.18**	*0.48*	1.03
876	F1R4Z2	8	Arrestin 3a, retinal (X-arrestin)	**2.77**	*0.34*	0.93
704	Q6IQP5	27	Enolase 1, (Alpha)	**2.11**	*0.49*	1.03
878	F1R4Z2	15	Arrestin 3a, retinal (X-arrestin	**2.72**	*0.44*	1.20
654	Q6NYE1	6	Fibrinogen, B beta polypeptide	*0.23*	**2.95**	0.68
1314	E7FES0	13	Apolipoprotein A-Ib	*0.19*	***6.44***	1.22
1291	E7FES0	10	Apolipoprotein A-Ib	*0.33*	**2.01**	0.66
904	Q6NYE1	9	Fibrinogen, B beta polypeptide	*0.12*	**8.02**	0.96
794	X1WCG6	7	Unknown	*0.42*	**2.53**	1.05
250	Q5RG12	3	Heat shock protein 90-alpha 2	*0.18*	**4.39**	0.80

Protein spots were identified by mass spectrometry analysis.

Proteins which show a 2-fold expression change were considered to be differentially expressed. Fold differences in expression between normal, degenerated and regenerating retina are shown in the table. (Numbers in Bold = 2-fold or more upregulated; Numbers in *italics* = 0.5-fold or less downregulated). N = normal retina; D = degenerated retina; R = regenerating retina.
