# Supplementary material for: Comparison of proteomic profiles in the zebrafish retina during experimental degeneration and regeneration
Source: Sci Rep. 2017 Mar 16;7:44601. doi: 10.1038/srep44601 (PMC5353638; doi:10.1038/srep44601)
Supplement: Supplementary Tables [file srep44601-s1.pdf]

Comparison of proteomic profiles in the zebrafish retina during experimental degeneration and regeneration.

Authors**: Karen Eastlake, Wendy E. Heywood, Dhani Tracey-White, Erika Aquino, Emily Bliss, Gerardo R Vasta, Kevin Mills, Peng T Khaw, Mariya Moosajee , G. Astrid Limb[*].**

Supplementary Table 1: **Original protein list and abundancies as identified by Progenesis Software**

Accession	Peptides (unique)	Score	P value	Description	Expression fmol		
					Normal retina	Degenerated retina	Regenerating retina
ENO1_YEAST	432 (413)	2828.09	1.07	Enolase 1 OS Saccharomyces cerevisiae strain ATCC 204508 S288c GN ENO1 PE 1 SV 3	137000	81316.66	151936.2
E7F8F6	216 (56)	1494.21	1.68	E7F8F6 DANRE Uncharacterized protein OS Danio rerio GN atp1a3b PE 3 SV 1	6915.43	2963.762	4384.602
Q6IQJ2	181 (19)	1282.66	1.44	Q6IQJ2 DANRE Uncharacterized protein OS Danio rerio GN zgc 153264 PE 2 SV 1	3946.03	1657.197	3538.934
Q6P271	177 (16)	1238.58	1.97	Q6P271 DANRE ATPase Na K transporting alpha 3a polypeptide OS Danio rerio GN atp1a3a PE 2 SV 1	2576.49	1570.916	1399.205
B8A518	154 (1)	1217.15	5.85	B8A518 DANRE Uncharacterized protein OS Danio rerio GN tuba1c PE 3 SV 1	99.56	157.3053	47.11084
Q8AY63	156 (113)	1141.45	1.63	Q8AY63 DANRE Brain subtype creatine kinase OS Danio rerio GN ckbb PE 2 SV 1	37200	13835.97	30493.49
Q6NWK7	144 (0)	1121.75	1	Q6NWK7 DANRE Tuba1 protein OS Danio rerio GN tuba1a PE 2 SV 1	0	0	0
Q7ZVG7	109 (106)	1103.88	31.01	Q7ZVG7 DANRE Fibrinogen gamma polypeptide OS Danio rerio GN fgg PE 2 SV 1	3432.91	44784.84	2527.729
F1R4K1	153 (2)	1084.88	2.03	F1R4K1 DANRE Uncharacterized protein OS Danio rerio GN tubb4b PE 3 SV 1	319.74	108.7701	167.2467
F1QEH9	155 (7)	1070.45	1.67	F1QEH9 DANRE Uncharacterized protein OS Danio rerio GN zgc 123194 PE 2 SV 1	1015.41	380.2949	646.5256
E7F244	134 (0)	1029.85	1	E7F244 DANRE Uncharacterized protein OS Danio rerio GN si ch211 114n24 6 PE 3 SV 1	0	0	0
Q6NWJ5	127 (1)	986.46	2.2	Q6NWJ5 DANRE Tubulin alpha 6 OS Danio rerio GN tuba8l4 PE 2 SV 1	89.23	24.63773	70.73001
Q6NW90	138 (3)	974.78	4.48	Q6NW90 DANRE Tubulin beta 5 OS Danio rerio GN tubb5 PE 2 SV 1	186.86	96.18425	755.0591
B0R068	138 (23)	937.47	2.48	B0R068 DANRE Uncharacterized protein OS Danio rerio GN atp1a1b PE 3 SV 1	1585.41	1297.395	914.7302
Q9DGL6	127 (25)	824.88	2.43	Q9DGL6 DANRE ATPase Na K transporting alpha 1 polypeptide OS Danio rerio GN atp1a1a 1 PE 2 SV 1	1271.62	1338.557	963.2648
Q6P972	106 (8)	822.66	2.09	Q6P972 DANRE Tubulin alpha 2 OS Danio rerio GN tuba2 PE 2 SV 1	1052.15	452.6121	535.1872
F1QCY2	107 (1)	810.56	46.75	F1QCY2 DANRE Uncharacterized protein Fragment OS Danio rerio GN tuba8l3 PE 2 SV 1	19.96	69.35583	2.592477
Q6NYE1	90 (74)	798.83	11.19	Q6NYE1 DANRE Fibrinogen B beta polypeptide OS Danio rerio GN fgb PE 2 SV 1	3930.02	24819.79	3885.624
E7F8S4	89 (85)	788.6	3.58	E7F8S4 DANRE Uncharacterized protein OS Danio rerio GN LOC557518 PE 4 SV 1	18800	3183.474	13387.38
B8A5L6	93 (7)	719.02	11.04	B8A5L6 DANRE Uncharacterized protein OS Danio rerio GN fga PE 2 SV 1	207.81	1391.595	269.6814
E9QD59	95 (23)	718.56	1.75	E9QD59 DANRE Uncharacterized protein OS Danio rerio GN actb1 PE 2 SV 1	4318.94	4318.94	4318.94
F1R2V7	92 (90)	707.93	1.73	F1R2V7 DANRE ATP synthase subunit beta OS Danio rerio GN zgc 163069 PE 3 SV 1	21400	8374.402	13174.89
R4GE02	103 (6)	701.81	1.35	R4GE02 DANRE Uncharacterized protein OS Danio rerio GN si ch211 113a14 11 PE 3 SV 1	1021.67	548.0864	1290.852

A3KPR4	105 (102)	698.24	2.27	A3KPR4 DANRE Histone H4 OS Danio rerio GN si ch211 113a14 26 PE 3 SV 1	86200	29674.51	117936.5
F1QKY8	91 (1)	697.51	45.19	F1QKY8 DANRE Uncharacterized protein OS Danio rerio GN zgc 112335 PE 3 SV 1	1.9	1.061971	84.06427
Q0D274	82 (79)	696.41	4.17	Q0D274 DANRE ES1 protein mitochondrial OS Danio rerio GN es1 PE 2 SV 1	48800	7100.036	30812.23
Q6PE34	97 (5)	680.6	2.14	Q6PE34 DANRE Uncharacterized protein OS Danio rerio GN zgc 65894 PE 2 SV 1	724.76	388.1475	359.1113
Q08BA1	100 (96)	673.27	1.61	Q08BA1 DANRE ATP synthase subunit alpha OS Danio rerio GN atp5a1 PE 2 SV 1	25600	9648.767	16999.85
E9QCD1	87 (2)	663.24	2.11	E9QCD1 DANRE Uncharacterized protein OS Danio rerio GN fga PE 2 SV 1	189.47	242.2326	221.7949
Q6PC12	91 (28)	607.58	1.78	Q6PC12 DANRE Enolase 1 Alpha OS Danio rerio GN eno1a PE 2 SV 1	5352.18	2265.518	3203.728
B0S5Q5	93 (2)	575.36	1.94	B0S5Q5 DANRE ATPase Na K transporting alpha 2a polypeptide OS Danio rerio GN atp1a2a PE 2 SV 1	55.11	18.73317	30.24911
B8JKS7	93 (3)	573.19	1.14	B8JKS7 DANRE Uncharacterized protein OS Danio rerio GN atp1a1a 4 PE 3 SV 1	148.33	83.87752	137.8688
F1RCB6	75 (2)	558.87	3.74	F1RCB6 DANRE Uncharacterized protein OS Danio rerio GN zgc 86725 PE 3 SV 1	17.9	40.67046	31.02473
Q7T334	67 (64)	556.03	1.97	Q7T334 DANRE Malate dehydrogenase OS Danio rerio GN mdh2 PE 2 SV 1	15500	4752.546	8621.783
Q8JHI0	86 (37)	540	1.59	Q8JHI0 DANRE Solute carrier family 25 alpha member 5 OS Danio rerio GN slc25a5 PE 2 SV 1	9026.63	3436.618	6146.244
Q6NYR4	71 (16)	529.05	1.21	Q6NYR4 DANRE Heat shock cognate 71 kDa protein OS Danio rerio GN hspa8 PE 2 SV 1	1297.72	951.9692	1405.813
Q6ZM17	70 (67)	520.16	3.07	Q6ZM17 DANRE Hemoglobin subunit alpha OS Danio rerio GN si xx by187g17 1 PE 2 SV 1	8953.8	14078.7	8035.416
F1Q766	81 (53)	513.7	3.12	F1Q766 DANRE Uncharacterized protein OS Danio rerio GN ckmt2a PE 2 SV 1	17400	3379.302	13281.13
Q6PC89	72 (22)	495.44	2.28	Q6PC89 DANRE Uncharacterized protein OS Danio rerio GN eno1b PE 2 SV 1	2233.21	593.8361	1326.137
Q1LWN2	60 (4)	494.48	46.66	Q1LWN2 DANRE Uncharacterized protein OS Danio rerio GN vtg1 PE 2 SV 1	63.06	542.4974	20.35732
E7EXF4	78 (4)	487.67	1.47	E7EXF4 DANRE Uncharacterized protein OS Danio rerio GN CR762436 3 PE 4 SV 1	140.86	85.00625	101.9672
F1QF78	74 (0)	476.51	1	F1QF78 DANRE Histone H2B OS Danio rerio GN zgc 171759 PE 3 SV 1	0	0	0
E7EZA5	74 (1)	469.08	2.44	E7EZA5 DANRE Uncharacterized protein OS Danio rerio GN CU302436 6 PE 4 SV 1	44.41	11.03236	31.0991
G1K2M4	75 (2)	468.75	8.65	G1K2M4 DANRE Histone H2B OS Danio rerio GN si ch211 113a14 22 PE 3 SV 1	38.43	2.694373	31.24785
F1QIH4	67 (1)	468.69	2.94	F1QIH4 DANRE Histone H2B OS Danio rerio GN si ch211 113a14 15 PE 3 SV 1	7.63	13.61144	17.37172
F1R3D3	67 (66)	462.22	1.71	F1R3D3 DANRE Glyceraldehyde 3 phosphate dehydrogenase OS Danio rerio GN gapdhs PE 3 SV 1	15200	5405.713	9475.218
Q6PC95	70 (1)	458.35	1.5	Q6PC95 DANRE Tubulin alpha 8 like 2 OS Danio rerio GN tuba8l2 PE 2 SV 1	41.26	28.80066	65.78943
E9QJ96	66 (8)	453.49	1.16	E9QJ96 DANRE 14 3 3 protein beta alpha A OS Danio rerio GN ywhaba PE 2 SV 1	1204.91	803.9426	1215.266
Q6DHS1	68 (13)	447.36	2.09	Q6DHS1 DANRE Actin alpha 2 smooth muscle aorta OS Danio rerio GN acta2 PE 2 SV 1	2067.11	2344.657	1965.14
B8JKS9	68 (2)	439.23	7.08	B8JKS9 DANRE Uncharacterized protein OS Danio rerio GN atp1a1a 2 PE 3 SV 1	39.33	61.14527	15.11924
E9QFD8	52 (0)	416.95	1	E9QFD8 DANRE Uncharacterized protein OS Danio rerio GN vtg4 PE 2 SV 1	0	0	0
F1R887	52 (1)	409.85	16.47	F1R887 DANRE Uncharacterized protein OS Danio rerio GN vtg4 PE 2 SV 1	13.46	1.165134	33.59596

F1QGY1	64 (1)	409.73	2.59	F1QGY1 DANRE Histone H2B OS Danio rerio GN zgc 171759 PE 3 SV 1	25.68	6.013792	21.54731
F1R2W8	54 (2)	404.15	1.25	F1R2W8 DANRE Uncharacterized protein Fragment OS Danio rerio GN si ch211 202a12 4 PE 2 SV 1	214.04	121.0829	181.3353
F1QV15	51 (2)	398.02	52.85	F1QV15 DANRE Uncharacterized protein Fragment OS Danio rerio GN vtg6 PE 4 SV 1	1.43	45.93177	1.827484
F1RBF6	56 (10)	396.58	1.72	F1RBF6 DANRE 14 3 3 protein beta alpha B OS Danio rerio GN ywhabb PE 3 SV 1	683.58	605.6938	617.8065
E7EXT3	57 (33)	390.04	1.62	E7EXT3 DANRE Phosphorylase OS Danio rerio GN pygmb PE 3 SV 1	3858.3	1537.668	2530.407
F1QTW6	50 (3)	381.21	1.45	F1QTW6 DANRE Uncharacterized protein OS Danio rerio GN vtg5 PE 4 SV 1	99.6	58.44483	73.20561
E7FES0	32 (28)	358.43	15.95	E7FES0 DANRE Uncharacterized protein OS Danio rerio GN apoa1b PE 3 SV 1	788.72	5522.894	606.4166
Q3B7P7	54 (2)	352.88	3.92	Q3B7P7 DANRE Ubiquitin A 52 residue ribosomal protein fusion product 1 OS Danio rerio GN uba52 PE 2	34.97	46.04707	145.6569
G1K2L0	52 (1)	350.13	10.99	G1K2L0 DANRE Histone H2B OS Danio rerio GN si ch211 113a14 28 PE 3 SV 1	9.63	45.87109	7.309937
F1R9V3	45 (2)	339.09	1.72	F1R9V3 DANRE Uncharacterized protein OS Danio rerio GN hspa8l PE 3 SV 1	36.17	37.80618	62.85695
B8JKT0	53 (1)	332.56	2.42	B8JKT0 DANRE Uncharacterized protein OS Danio rerio GN atp1a1a 3 PE 3 SV 1	81.56	20.48694	40.20465
Q803M8	53 (20)	329.69	1.74	Q803M8 DANRE Tryosine 3 monooxygenase tryptophan 5 monooxygenase activation protein zeta polypeptid	1429.12	685.6815	872.9424
E9QBP3	47 (1)	328.73	1.31	E9QBP3 DANRE Uncharacterized protein OS Danio rerio GN actb1 PE 2 SV 1	23.07	18.37514	28.15601
E7F4R9	38	326.6	2.2	E7F4R9 DANRE Isocitrate dehydrogenase NADP OS Danio rerio PE 3 SV 1	8112.66	2237.865	5259.149
F1Q8F1	37 (23)	323.55	1.84	F1Q8F1 DANRE Uncharacterized protein OS Danio rerio GN inab PE 3 SV 1	2874.2	1263.819	4068.936
F1QC15	45 (1)	321.49	3.45	F1QC15 DANRE Uncharacterized protein OS Danio rerio GN actb2 PE 2 SV 1	24.15	11.52997	7.437435
Q6NYQ7	42 (41)	321.26	2.15	Q6NYQ7 DANRE Nicotinamide nucleotide transhydrogenase OS Danio rerio GN nnt PE 2 SV 1	6464.18	2361.806	3201.274
F1R4Z1	41 (40)	320.99	3.32	F1R4Z1 DANRE Uncharacterized protein Fragment OS Danio rerio GN arr3a PE 4 SV 1	9049.65	1651.96	8827.545
F8W4M7	37	319.89	2.19	F8W4M7 DANRE Uncharacterized protein Fragment OS Danio rerio GN aco2 PE 4 SV 2	5099.46	1411.408	2953.586
Q6PGX4	44 (2)	319.06	2.01	Q6PGX4 DANRE Heat shock cognate 71 kDa protein OS Danio rerio GN hsc70 PE 2 SV 1	163.3	49.41504	106.4616
Q9DDU5	45 (31)	318.78	1.44	Q9DDU5 DANRE Glutathione S transferase pi OS Danio rerio GN gstp1 PE 2 SV 1	4428.71	1945.119	3264.854
Q7ZU62	49 (1)	309.41	2.01	Q7ZU62 DANRE Uncharacterized protein Fragment OS Danio rerio GN rps27a PE 2 SV 1	242.77	99.76461	128.2851
B0R193	48 (1)	309.21	1.45	B0R193 DANRE Uncharacterized protein OS Danio rerio GN ubb PE 4 SV 1	18.13	7.591577	16.11799
E9QBV1	39 (8)	307.88	2.33	E9QBV1 DANRE Clathrin heavy chain OS Danio rerio GN cltcb PE 2 SV 1	440.64	136.1386	200.5239
F1QVD3	46	304.22	1.56	F1QVD3 DANRE Uncharacterized protein OS Danio rerio GN rlbp1a PE 2 SV 1	9699.62	4095.598	6614.048
Q7ZUM0	46 (6)	302.15	1.53	Q7ZUM0 DANRE Tyrosine 3 monooxygenase tryptophan 5 monooxygenase activation protein theta polypepti	418.65	229.9744	291.08
Q6TH14	44 (1)	300.21	1.58	Q6TH14 DANRE Enolase 1 Alpha OS Danio rerio GN eno3 PE 2 SV 1	36.82	24.55278	27.24226
F1R6T2	43 (1)	299.92	1.19	F1R6T2 DANRE Uncharacterized protein OS Danio rerio GN tuba1b PE 2 SV 1	66.33	38.46156	59.32948

A8KBJ5	58 (38)	285.03	2.19	A8KBJ5 DANRE Histone H3 OS Danio rerio GN si ch211 113a14 23 PE 2 SV 1	29800	8253.034	29962.24
Q6TNP9	41 (0)	282.49	1	Q6TNP9 DANRE Tubulin alpha 2 OS Danio rerio GN tuba8l PE 2 SV 1	0	0	0
Q08BV6	37 (1)	277.05	1.52	Q08BV6 DANRE Uncharacterized protein OS Danio rerio GN ckba PE 2 SV 1	23.66	10.52869	16.53236
Q6NX10	44 (2)	275.77	2.93	Q6NX10 DANRE Solute carrier family 25 Mitochondrial carrier adenine nucleotide translocator membe	208.69	143.445	85.75363
Q6P102	43 (12)	275.74	1.19	Q6P102 DANRE Tyrosine 3 monooxygenase tryptophan 5 monooxygenase activation protein beta polypeptid	1024.73	638.3236	939.6881
B3DFZ2	47 (2)	275.36	1.8	B3DFZ2 DANRE Atp1a1a 5 protein OS Danio rerio GN atp1a1a 5 PE 2 SV 1	151.17	154.2104	149.9068
F1R966	35 (3)	274.99	2.57	F1R966 DANRE Clathrin heavy chain OS Danio rerio GN cltca PE 3 SV 1	92.59	62.77767	42.7015
Q90WX5	38 (27)	274.33	3.13	Q90WX5 DANRE Cone transducin alpha subunit OS Danio rerio GN gnat2 PE 2 SV 1	4224.81	819.9935	2772.166
Q7ZW20	41 (12)	274.22	1.33	Q7ZW20 DANRE Tyrosine 3 monooxygenase tryptophan 5 monooxygenase activation protein epsilon polypep	472.1	245.5581	377.1417
Q6TGS5	46 (0)	273.82	1	Q6TGS5 DANRE Alpha tubulin like protein OS Danio rerio GN tuba4l PE 2 SV 1	0	0	0
B3DH85	40	270.69	1.4	B3DH85 DANRE NCAM OS Danio rerio GN ncam1a PE 2 SV 1	4605.29	2216.54	3488.093
Q801E6	36 (34)	267.91	1.65	Q801E6 DANRE Malate dehydrogenase OS Danio rerio GN mdh1aa PE 2 SV 1	5726.41	2105.161	4464.82
F1R7H5	56 (12)	255.34	1.52	F1R7H5 DANRE Histone H2A Fragment OS Danio rerio GN zgc 195633 PE 3 SV 1	3652.84	1457.103	3076.485
Q9I8N9	36 (30)	254.99	4.54	Q9I8N9 DANRE Brain type fatty acid binding protein OS Danio rerio GN fabp7a PE 2 SV 1	1579.89	1036.532	7618.569
B0S730	41 (4)	251.09	2.69	B0S730 DANRE Solute carrier family 25 Mitochondrial carrier adenine nucleotide translocator membe	456.21	242.6574	180.4853
E7EZ16	37 (22)	250.76	1.43	E7EZ16 DANRE Uncharacterized protein OS Danio rerio GN hsp90ab1 PE 3 SV 1	1603.96	1389.993	1939.981
Q6GQM9	30 (8)	241.17	1.96	Q6GQM9 DANRE Enolase 2 OS Danio rerio GN eno2 PE 2 SV 1	649.52	464.5973	415.6676
Q7SYK7	33 (23)	235.47	2.78	Q7SYK7 DANRE Aspartate aminotransferase OS Danio rerio GN got2a PE 2 SV 1	3557.09	776.9806	2343.196
Q7T3G2	36 (2)	234.88	1.29	Q7T3G2 DANRE Tyrosine 3 monooxygenase tryptophan 5 monooxygenase activation protein eta polypeptide	121.48	92.54928	166.9067
F1R2T3	29 (0)	231.11	1	F1R2T3 DANRE Uncharacterized protein OS Danio rerio GN vtg7 PE 4 SV 1	0	0	0
E7F354	36 (2)	226.72	1.57	E7F354 DANRE Uncharacterized protein OS Danio rerio GN ywhag2 PE 4 SV 1	228.26	88.50772	183.0565
Q66I80	27 (26)	225.33	5.03	Q66I80 DANRE Fatty acid binding protein 11a OS Danio rerio GN fabp11a PE 2 SV 1	1994.21	4494.457	1565.315
F8W3W8	26 (7)	210.63	3.93	F8W3W8 DANRE Uncharacterized protein OS Danio rerio GN mbpa PE 2 SV 1	1454.9	1222.117	544.399
A2ATX5	27 (12)	210.24	1.76	A2ATX5 DANRE Uncharacterized protein OS Danio rerio GN inaa PE 3 SV 1	2076.04	715.3135	1371.814
B0UXR9	31 (5)	202.91	1.83	B0UXR9 DANRE Uncharacterized protein OS Danio rerio GN mcm5 PE 3 SV 1	285.87	192.6537	184.7778
Q5CZQ1	29 (2)	196.42	2.73	Q5CZQ1 DANRE Tyrosine 3 monooxygenase tryptophan 5 monooxygenase activation protein epsilon polypep	54.27	16.56675	21.11169
Q7T2P7	25 (16)	195.96	1.68	Q7T2P7 DANRE Glutamine synthetase OS Danio rerio GN glula PE 2 SV 1	4442.82	2197.704	2815.824

Q6PC86	34 (5)	194.86	4.24	Q6PC86 DANRE Creatine kinase mitochondrial 2 Sarcomeric OS Danio rerio GN ckmt2b PE 2 SV 1	638.09	91.26884	402.7265
Q6P3J5	26	194.77	2.34	Q6P3J5 DANRE Eukaryotic translation elongation factor 2 OS Danio rerio GN eef2b PE 2 SV 1	1378.61	1958.572	2446.066
F1QV28	29 (2)	184.76	2.12	F1QV28 DANRE Uncharacterized protein OS Danio rerio GN zgc 112515 PE 3 SV 1	162.77	58.08679	215.3244
Q6P025	22	183.93	3.48	Q6P025 DANRE Gnb3 protein OS Danio rerio GN gnb3b PE 2 SV 1	3228.1	562.7962	1756.871
G1K2S9	42 (25)	183.28	1.92	G1K2S9 DANRE Histone H3 OS Danio rerio GN h3f3b 1 PE 2 SV 1	13500	4262.267	12112.39
B7ZV62	25 (22)	182.19	2.46	B7ZV62 DANRE N ethylmaleimide sensitive factor OS Danio rerio GN nsfa PE 2 SV 1	3244.75	799.1	1836.452
Q5RG12	26 (11)	178.93	2.15	Q5RG12 DANRE Heat shock protein 90 alpha 2 OS Danio rerio GN hsp90aa1 2 PE 2 SV 1	904.4	254.6789	767.8727
B8JLJ3	30 (1)	178.82	3.43	B8JLJ3 DANRE Uncharacterized protein OS Danio rerio GN ywhaz PE 3 SV 1	15.08	3.889849	4.674959
F1QXV8	21 (19)	178.09	1.92	F1QXV8 DANRE Phosphoglycerate kinase OS Danio rerio GN pgk1 PE 2 SV 1	3714.93	1174.722	2138.666
Q6DGJ6	18 (17)	178.08	1.63	Q6DGJ6 DANRE Peroxiredoxin 2 OS Danio rerio GN prdx2 PE 2 SV 1	2430.92	903.5919	1911.548
Q7ZU59	42 (18)	177.64	1.61	Q7ZU59 DANRE Histone H2A Fragment OS Danio rerio GN h2afvb PE 2 SV 1	3612.21	1414.358	3980.239
E7FC32	26 (4)	174.39	1.23	E7FC32 DANRE Uncharacterized protein OS Danio rerio GN tubb1 PE 3 SV 1	422.24	263.4478	553.5046
Q503C7	23 (2)	174.05	1.35	Q503C7 DANRE Phosphorylase OS Danio rerio GN pygma PE 2 SV 1	83.99	41.45936	65.93818
F1QM13	17	173.22	2.2	F1QM13 DANRE Uncharacterized protein OS Danio rerio GN stxbp1a PE 4 SV 1	1812.36	741.353	876.8418
B2GTW6	23 (22)	170.33	1.62	B2GTW6 DANRE Citrate synthase OS Danio rerio GN cs PE 2 SV 1	3736.94	1722.584	2446.736
Q6NX09	19	169.44	1.96	Q6NX09 DANRE Hexokinase 1 OS Danio rerio GN hk1 PE 2 SV 1	2902.47	1169.036	1574.771
A2BEN1	26 (8)	168.31	1.32	A2BEN1 DANRE Uncharacterized protein OS Danio rerio GN atp6v1aa PE 3 SV 1	529.61	243.0883	450.783
E7F5U9	24	168.03	2.04	E7F5U9 DANRE Pyruvate kinase OS Danio rerio GN pkma PE 2 SV 1	4585.77	1808.361	2388.416
E7FA49	22	162.96	1.74	E7FA49 DANRE L lactate dehydrogenase OS Danio rerio GN ldhbb PE 2 SV 1	7900.35	2759.463	4828.606
E9QBF0	18 (11)	162.55	1.57	E9QBF0 DANRE Triosephosphate isomerase OS Danio rerio GN tpi1b PE 2 SV 1	2316.59	896.686	1738.129
A5PMG2	23 (2)	149.97	1.57	A5PMG2 DANRE Uncharacterized protein OS Danio rerio GN si ch211 199o1 2 PE 2 SV 1	74.65	51.96984	58.04387
F8W3M5	17 (2)	144.66	1.29	F8W3M5 DANRE Uncharacterized protein OS Danio rerio GN mbpa PE 2 SV 2	545.46	405.4242	552.1765
Q6DRD1	22 (19)	142.05	2.09	Q6DRD1 DANRE ATP synthase oligomycin sensitivity conferral protein OS Danio rerio GN atp5o PE 2 SV 1	3340.45	968.3115	1864.937
Q8AWD0	20 (14)	135.31	2.25	Q8AWD0 DANRE Uncharacterized protein OS Danio rerio GN vdac2 PE 2 SV 1	1851.47	768.1997	875.3012
Q6PC38	18 (17)	130.47	3.83	Q6PC38 DANRE Recoverin OS Danio rerio GN rcv1 PE 2 SV 1	1971.44	312.4077	1732.784
F1QCE3	21	130.02	1.36	F1QCE3 DANRE Uncharacterized protein OS Danio rerio GN prdx5 PE 2 SV 1	1586.87	708.5412	1404.092
Q6P3L3	19 (11)	129.59	3.28	Q6P3L3 DANRE Heat shock protein 5 OS Danio rerio GN hspa5 PE 2 SV 1	436.48	869.5178	554.3652
E7FCD8	19 (1)	124.41	1.64	E7FCD8 DANRE Uncharacterized protein OS Danio rerio GN atp6v1ab PE 3 SV 1	14.83	11.79698	25.79728

E9QEB6	15 (3)	123.32	1.7	E9QEB6 DANRE Uncharacterized protein OS Danio rerio GN arf1l PE 2 SV 1	216.09	85.91651	135.1382
F1RBS6	15	123.29	1.99	F1RBS6 DANRE Nucleoside diphosphate kinase OS Danio rerio GN nme2b 2 PE 2 SV 1	642.19	775.2451	861.4356
E7FD96	13 (9)	122.58	1.36	E7FD96 DANRE Elongation factor 1 alpha OS Danio rerio GN eef1a1l1 PE 2 SV 1	2133.45	1438.704	1855.077
B3DFP9	16 (13)	121.81	12.73	B3DFP9 DANRE Uncharacterized protein OS Danio rerio GN apoa2 PE 2 SV 1	480.78	2939.688	404.2777
B8JIS1	12	121.26	1.87	B8JIS1 DANRE Uncharacterized protein OS Danio rerio GN atp5f1 PE 4 SV 1	2084.08	677.1857	1263.227
F1QCD4	14 (8)	112.69	2.02	F1QCD4 DANRE Aspartate aminotransferase OS Danio rerio GN got2b PE 3 SV 1	1498.19	449.8146	867.46
Q7ZV39	13 (9)	112.64	2.5	Q7ZV39 DANRE Uncharacterized protein OS Danio rerio GN zgc 56235 PE 2 SV 1	1094.72	473.6331	465.796
E7FBE9	16 (15)	108.35	3.19	E7FBE9 DANRE Nucleoside diphosphate kinase OS Danio rerio GN nme2a PE 2 SV 1	2424.55	461.6177	1667.197
Q6P603	12	108.22	11.04	Q6P603 DANRE Annexin OS Danio rerio GN anxa2a PE 2 SV 1	221.13	1481.165	738.6967
Q6PC53	15	108.13	1.52	Q6PC53 DANRE Peptidyl prolyl cis trans isomerase OS Danio rerio GN ppiab PE 2 SV 1	3025.93	1208.541	2771.072
Q803H5	10 (6)	107.86	2.09	Q803H5 DANRE Gnb1l protein OS Danio rerio GN gnb1b PE 2 SV 1	1399.92	611.5134	710.3495
E7F5N5	10	105.44	1.78	E7F5N5 DANRE Uncharacterized protein OS Danio rerio GN LOC798555 PE 4 SV 1	1562.43	747.6824	934.8963
Q5RH26	10	105.14	1.96	Q5RH26 DANRE ATP synthase subunit gamma OS Danio rerio GN atp5c1 PE 3 SV 1	1761.19	583.1315	956.1567
Q6TH32	21 (20)	104.61	1.1	Q6TH32 DANRE Muscle cofilin 2 OS Danio rerio GN cfl1 PE 2 SV 1	2270.48	1291.503	2202.087
Q6PBP1	13 (8)	103.78	1.78	Q6PBP1 DANRE Guanine nucleotide binding protein G protein alpha activating activity polypeptide O	882.8	413.5377	527.1017
Q6NWC1	17 (15)	102.39	1.29	Q6NWC1 DANRE Uncharacterized protein OS Danio rerio GN vdac1 PE 2 SV 1	3272.46	1540.38	2961.396
F1R9C4	13 (6)	102.31	1.8	F1R9C4 DANRE Uncharacterized protein Fragment OS Danio rerio GN atp2b1b PE 3 SV 1	489.35	175.7471	289.125
Q78AP9	13 (1)	99.88	2.11	Q78AP9 DANRE ADP ribosylation factor 3b OS Danio rerio GN arf3b PE 2 SV 1	12.8	4.090106	15.11924
Q6P6E0	10	95.22	1.67	Q6P6E0 DANRE ATP synthase H transporting mitochondrial F0 complex subunit g OS Danio rerio GN at	1658.62	602.9327	1195.175
E7EXJ0	12 (4)	94.61	1.88	E7EXJ0 DANRE Uncharacterized protein OS Danio rerio GN gnai2a PE 4 SV 1	519.65	185.1228	294.0443
Q7ZUW4	13 (2)	93.65	1.34	Q7ZUW4 DANRE ADP ribosylation factor 5 OS Danio rerio GN arf5 PE 2 SV 1	31.39	20.4566	26.64727
Q7SZZ5	13 (6)	91.88	1.86	Q7SZZ5 DANRE Uncharacterized protein OS Danio rerio GN vamp2 PE 4 SV 1	1806.63	588.8418	1078.662
Q50LC6	14 (1)	91.43	5.11	Q50LC6 DANRE Glutathione S transferase pi 2 OS Danio rerio GN gstp2 PE 2 SV 1	7.15	15.16495	5.19558
Q5TZD2	12	91.22	2.31	Q5TZD2 DANRE Uncharacterized protein OS Danio rerio GN prdx6 PE 4 SV 1	1220.1	1142.438	866.8437
Q90WX6	11 (6)	90.03	2.22	Q90WX6 DANRE Guanine nucleotide binding protein G protein alpha transducing activity polypeptide	477.05	130.4404	386.3854
Q6P981	10	88.72	3.32	Q6P981 DANRE Opn1sw1 protein OS Danio rerio GN opn1sw1 PE 2 SV 1	2166.2	395.4842	1284.7
E9QDI1	12 (10)	87.59	9.35	E9QDI1 DANRE Uncharacterized protein OS Danio rerio GN apoc1l PE 2 SV 1	405.18	1573.04	294.5224
F1QCV3	16 (3)	87.12	1.5	F1QCV3 DANRE Glutamine synthetase OS Danio rerio GN glulb PE 2 SV 1	238.62	120.6703	169.1485
Q52JI4	10	86.73	6.56	Q52JI4 DANRE Beta B2 crystallin OS Danio rerio GN crybb2 PE 2 SV 1	622.42	1407.949	376.0367

Q7ZTT0	34 (7)	83.42	1.66	Q7ZTT0 DANRE Histone H2A OS Danio rerio GN phc2b PE 2 SV 1	7792.33	3647.222	10608.32
Q6DH91	16 (1)	82.4	4.15	Q6DH91 DANRE Histone H2B OS Danio rerio GN zgc 92591 PE 3 SV 1	511.68	751.906	2257.272
Q6PHI8	10	80.52	1.4	Q6PHI8 DANRE Tkt protein OS Danio rerio GN tktb PE 2 SV 1	652.58	328.5618	803.9549
I3ISI4	10 (4)	80.13	2.64	I3ISI4 DANRE Uncharacterized protein OS Danio rerio GN fabp3 PE 2 SV 1	324.22	258.1379	910.8627
Q6P969	8 (2)	76.72	6.33	Q6P969 DANRE Elongation factor 1 alpha OS Danio rerio GN eef1a1a PE 2 SV 1	202.48	36.07668	33.96783
U3JAS0	10	76.19	2.65	U3JAS0 DANRE Synaptosomal associated protein OS Danio rerio GN snap25a PE 3 SV 1	1395.28	319.3074	636.347
F8W3A5	7	73.64	2.75	F8W3A5 DANRE Uncharacterized protein OS Danio rerio GN dnm1a PE 3 SV 1	1063.75	234.9566	694.2846
A3KPR3	9 (8)	68.59	2.38	A3KPR3 DANRE Uncharacterized protein OS Danio rerio GN histh1l PE 3 SV 1	1810.28	523.8977	2179.583
E7FDP2	7 (3)	66.87	1.35	E7FDP2 DANRE 6 phosphofructokinase OS Danio rerio GN pfkpa PE 2 SV 1	849.93	624.7061	812.2848
B8JL43	8 (7)	65	9.2	B8JL43 DANRE Uncharacterized protein OS Danio rerio GN tfa PE 2 SV 1	103.08	575.4185	126.3408
Q1MTI1	6	64.11	1.63	Q1MTI1 DANRE Uncharacterized protein OS Danio rerio GN stx1b PE 3 SV 1	814.68	418.6958	532.2973
E9QFJ1	7 (3)	62.99	2.69	E9QFJ1 DANRE 6 phosphofructokinase OS Danio rerio GN pfkpb PE 3 SV 1	312.44	73.36097	123.2596
I3IS66	7 (2)	62.72	2.04	I3IS66 DANRE Triosephosphate isomerase A Fragment OS Danio rerio GN tpi1a PE 2 SV 1	138.58	43.82604	72.20687
Q7T368	8 (7)	61.49	2.49	Q7T368 DANRE Pyruvate dehydrogenase Lipoamide beta OS Danio rerio GN pdhb PE 2 SV 1	985.43	287.776	419.8432
F1QDG2	10 (9)	61.24	2.12	F1QDG2 DANRE Uncharacterized protein OS Danio rerio GN ompa PE 4 SV 1	1101.14	314.7197	917.4077
F1R5X8	6	61.22	3.18	F1R5X8 DANRE Uncharacterized protein OS Danio rerio GN vdac3 PE 2 SV 1	962.72	592.8166	325.9722
B8JKN6	9	60.11	1.35	B8JKN6 DANRE Peptidyl prolyl cis trans isomerase OS Danio rerio GN ppiaa PE 2 SV 1	746.9	496.2804	1072.956
F1R2R5	8 (1)	59.62	2.43	F1R2R5 DANRE Uncharacterized protein OS Danio rerio GN atp2b3a PE 3 SV 1	69.73	19.81942	30.46161
Q4V914	6	57.9	3.6	Q4V914 DANRE Histone H2A OS Danio rerio GN h2afy2 PE 2 SV 1	976.83	164.8665	573.6281
F1RC82	7	57.83	1.96	F1RC82 DANRE Uncharacterized protein Fragment OS Danio rerio GN pvrl1b PE 4 SV 1	1084.04	343.0167	587.5468
E7FAV1	7 (6)	57.51	2.25	E7FAV1 DANRE Uncharacterized protein OS Danio rerio GN btbd17b PE 4 SV 1	874.8	316.4189	412.6714
F1R5T1	7	57.34	2.88	F1R5T1 DANRE Uncharacterized protein Fragment OS Danio rerio GN slc12a5b PE 4 SV 1	873.64	252.7431	322.5403
Q7T3D3	6 (1)	56.37	2.66	Q7T3D3 DANRE Guanine nucleotide binding protein G protein alpha inhibiting activity polypeptide 1	24.31	19.65557	12.91989
Q9DGL2	5	55.45	1.77	Q9DGL2 DANRE ATPase Na K transporting beta 2a polypeptide OS Danio rerio GN atp1b2a PE 2 SV 1	670.22	364.2743	402.5778
F1Q5F3	7 (5)	55.43	2.1	F1Q5F3 DANRE Uncharacterized protein OS Danio rerio GN lmnb1 PE 2 SV 1	388.18	131.4902	483.752
E7F0A1	5	55.13	3.02	E7F0A1 DANRE Uncharacterized protein OS Danio rerio GN pfn1 PE 4 SV 1	430.3	788.1465	490.8495
B8A4H6	6	54.83	2.22	B8A4H6 DANRE Uncharacterized protein OS Danio rerio GN pgam1b PE 3 SV 1	1302.67	439.3587	623.034
Q4V8S5	7	54.2	1.39	Q4V8S5 DANRE Acbd7 protein OS Danio rerio GN acbd7 PE 4 SV 1	556.7	248.5559	426.0482
F1R319	7	53.47	2.05	F1R319 DANRE Uncharacterized protein OS Danio rerio GN slc25a11 PE 3 SV 1	594.35	200.039	308.4517

Q6PBJ3	7 (1)	52.24	3.62	Q6PBJ3 DANRE Uncharacterized protein OS Danio rerio GN vamp1 PE 4 SV 1	31.77	24.96543	12.0699
O93548	8	50.86	3.79	O93548 DANRE Embryonic 1 beta globin OS Danio rerio GN bE1 PE 2 SV 1	1277.63	2273.814	1050.973
Q6PC92	7	50.78	2.96	Q6PC92 DANRE ADP ribosylation factor like 3 like 1 OS Danio rerio GN arl3l1 PE 2 SV 1	736.1	151.1094	618.0084
E7FBA5	5 (2)	50.51	3.44	E7FBA5 DANRE Uncharacterized protein OS Danio rerio GN arf4a PE 3 SV 1	145.18	231.0667	117.639
F8W246	8	50.28	1.43	F8W246 DANRE Uncharacterized protein OS Danio rerio GN rps13 PE 4 SV 1	331.58	155.3452	389.4241
F1QLR1	7 (4)	48.76	1.48	F1QLR1 DANRE Uncharacterized protein OS Danio rerio GN cox4i2 PE 4 SV 1	308.1	125.9741	310.3642
F1QTL9	7	48.61	3.3	F1QTL9 DANRE Uncharacterized protein OS Danio rerio GN hnrnpa0b PE 4 SV 1	779.71	165.4248	956.8261
B8JL30	9 (8)	48.44	2.02	B8JL30 DANRE Uncharacterized protein OS Danio rerio GN hmgb2a PE 2 SV 1	883.94	266.0632	778.8589
B0S5D8	8 (7)	48.16	1.81	B0S5D8 DANRE Uncharacterized protein OS Danio rerio GN zc3h13 PE 2 SV 1	748.48	272.4229	440.1156
D2K290	5 (4)	45.67	11.15	D2K290 DANRE Complexin 3b OS Danio rerio GN cplx3b PE 2 SV 1	685.41	37.30857	148.175
E9QG44	5 (3)	44.94	1.53	E9QG44 DANRE Uncharacterized protein OS Danio rerio PE 3 SV 1	357.96	270.5963	580.7362
Q6XG62	6	44.82	3.67	Q6XG62 DANRE Ictacalcin OS Danio rerio GN icn PE 4 SV 1	162.48	196.8834	633.1808
Q6NYU8	5 (2)	43.33	2.23	Q6NYU8 DANRE Heterogeneous nuclear ribonucleoprotein A B OS Danio rerio GN hnrnpaba PE 2 SV 1	352.89	95.95972	292.9925
Q803Q7	5	43.22	1.46	Q803Q7 DANRE Fructose bisphosphate aldolase OS Danio rerio GN aldoaa PE 2 SV 1	751.58	346.0084	548.2134
Q4VBU7	6	43.16	1.99	Q4VBU7 DANRE Cytochrome c oxidase subunit Vaa OS Danio rerio GN cox5aa PE 2 SV 1	346.77	130.58	454.5229
F1QAM8	5 (3)	42.46	4.19	F1QAM8 DANRE Uncharacterized protein OS Danio rerio GN vim PE 3 SV 1	112.23	39.68131	290.9206
Q6TNV0	5 (3)	42.35	1.53	Q6TNV0 DANRE Cytochrome c oxidase subunit IV isoform 1 OS Danio rerio GN cox4i1 PE 2 SV 1	140.78	130.58	152.4674
F1REN2	5	42.19	2.67	F1REN2 DANRE Uncharacterized protein OS Danio rerio GN slc25a3a PE 2 SV 1	1214.97	366.0585	482.9871
Q7T306	8 (1)	41.87	3.06	Q7T306 DANRE Ckmb protein OS Danio rerio GN ckmb PE 2 SV 1	24.14	6.177639	33.07534
F1QTU1	5	41.31	3.35	F1QTU1 DANRE Uncharacterized protein OS Danio rerio GN pde6c PE 4 SV 1	484.74	87.8584	418.377
I3ITF4	5	39.58	12	I3ITF4 DANRE Uncharacterized protein OS Danio rerio GN zgc 73075 PE 2 SV 1	511.46	32.37495	680.1641
F1R314	4 (2)	39.14	1.7	F1R314 DANRE Uncharacterized protein OS Danio rerio GN zgc 110425 PE 3 SV 1	165.95	130.7135	299.9518
Q6DHE8	6 (1)	37.78	1.55	Q6DHE8 DANRE Ras homolog gene family member Ad OS Danio rerio GN rhoad PE 2 SV 1	13.11	8.465428	9.562417
Q6NW56	5	37.78	2.78	Q6NW56 DANRE KH domain containing RNA binding signal transduction associated 1 OS Danio rerio GN k	1118.53	244.5507	669.6348
Q6NUX8	6 (1)	37.72	1.4	Q6NUX8 DANRE Ras homolog gene family member Aa OS Danio rerio GN rhoaa PE 2 SV 1	42.01	18.16274	33.95721
E7FAZ5	5	37.54	5.93	E7FAZ5 DANRE Uncharacterized protein OS Danio rerio GN hp1bp3 PE 3 SV 1	578.9	59.27013	295.3087
E7F1F5	4	36.86	6.4	E7F1F5 DANRE Guanine nucleotide binding protein subunit gamma OS Danio rerio GN gngt2b PE 3 SV 1	626.01	59.3733	279.6051
Q8UUT4	3	36.66	2.53	Q8UUT4 DANRE DMbeta1 OS Danio rerio GN gpm6aa PE 2 SV 1	583.31	345.3409	244.9679

Q803W0	3	36.54	1.91	Q803W0 DANRE DEAD Asp Glu Ala Asp box polypeptide 39b OS Danio rerio GN ddx39ab PE 2 SV 1	285.99	94.44262	316.4523
F1R0G9	5	36.45	1.5	F1R0G9 DANRE Uncharacterized protein OS Danio rerio GN slc25a12 PE 2 SV 1	442.89	362.296	423.8382
E9QJ49	4	35.93	2.05	E9QJ49 DANRE Uncharacterized protein OS Danio rerio GN slc25a3b PE 2 SV 1	329.24	363.7161	309.8967
Q6PH15	5	35.57	3.18	Q6PH15 DANRE Solute carrier family 1 Glial high affinity glutamate transporter member 2 OS Danio	367.47	406.7835	223.8456
Q6NX86	6	31.92	2.29	Q6NX86 DANRE High mobility group box 1 OS Danio rerio GN hmgb1a PE 2 SV 1	772.46	204.2808	459.0385
Q6PC77	7	31.74	2.58	Q6PC77 DANRE ATP synthase H transporting mitochondrial F0 complex subunit d OS Danio rerio GN at	918.16	216.1809	562.4826
Q6NYA1	3 (1)	31.67	2.42	Q6NYA1 DANRE Uncharacterized protein OS Danio rerio GN hnrnpabb PE 2 SV 1	260.71	65.49632	223.9412
Q5XTP0	3	31.33	12.59	Q5XTP0 DANRE Crystallin gamma S1 OS Danio rerio GN crygs1 PE 2 SV 1	86.35	363.6978	50.57456
Q801U8	5	31.23	2.22	Q801U8 DANRE Rhodopsin OS Danio rerio GN opn1mw2 PE 3 SV 1	1396.95	382.2004	866.0256
F1QZL6	4	30.81	2.84	F1QZL6 DANRE Glucose 6 phosphate isomerase OS Danio rerio GN gpia PE 3 SV 1	514.03	142.1828	192.3533
F1QCS6	3	29.57	1.76	F1QCS6 DANRE Uncharacterized protein Fragment OS Danio rerio GN LOC100537765 PE 3 SV 1	379.68	195.4513	229.4343
Q1MTC4	6 (3)	29.5	7.49	Q1MTC4 DANRE Uncharacterized protein OS Danio rerio GN vtg2 PE 2 SV 1	19.89	90.46781	21.21794
Q5BJB9	3	29.3	5.02	Q5BJB9 DANRE Prdx3 protein OS Danio rerio GN prdx3 PE 2 SV 1	605.84	73.18499	287.2231
Q5RGB5	4	29.28	2.19	Q5RGB5 DANRE Si ch211 255d18 4 OS Danio rerio GN rab3ab PE 2 SV 1	306.49	138.3597	148.7168
F1R8E1	3 (0)	28.83	1	F1R8E1 DANRE Uncharacterized protein Fragment OS Danio rerio GN ncor2 PE 4 SV 1	0	0	0
F1Q9A4	4	28.5	2.24	F1Q9A4 DANRE Uncharacterized protein OS Danio rerio GN cox7a2a PE 4 SV 1	329.8	107.6232	156.388
F1R6L4	4	27.11	5.19	F1R6L4 DANRE Uncharacterized protein Fragment OS Danio rerio GN cirbp PE 4 SV 1	566.26	117.3509	1066.454
F1REH8	2	25.54	1.38	F1REH8 DANRE Annexin OS Danio rerio GN anxa13 PE 2 SV 1	68.53	40.42166	52.76329
Q568Q7	3	25.13	3.12	Q568Q7 DANRE Stathmin OS Danio rerio GN stmn1b PE 2 SV 1	488.38	94.84314	476.3677
Q0ZBR7	4 (3)	24.93	1.42	Q0ZBR7 DANRE Macrophage migration inhibitory factor OS Danio rerio GN mif PE 4 SV 1	206.04	88.04652	153.7743
F1R3Q3	2	24.82	1.97	F1R3Q3 DANRE Uncharacterized protein OS Danio rerio GN sept6 PE 2 SV 1	161.23	49.62137	115.9177
H9GY51	3 (2)	24.49	2.28	H9GY51 DANRE Uncharacterized protein OS Danio rerio PE 4 SV 1	444.15	127.8613	207.2495
Q6DH01	2	24.1	1.94	Q6DH01 DANRE Retinoschisis X linked juvenile 1 OS Danio rerio GN rs1 PE 2 SV 1	854.89	266.9614	481.5208
Q6NUZ3	2 (1)	24.04	1.43	Q6NUZ3 DANRE Synaptotagmin binding cytoplasmic RNA interacting protein OS Danio rerio GN syncrip PE	90.42	45.1975	113.1128
F1QE30	3	23.78	1.92	F1QE30 DANRE Uncharacterized protein OS Danio rerio GN atp6v0a1b PE 4 SV 1	226.15	80.4064	125.1083
B3DFN3	4 (3)	23.71	2.26	B3DFN3 DANRE Lamin B2 OS Danio rerio GN lmnb2 PE 2 SV 1	216.11	83.75616	331.7734
Q803J2	4	23.6	1.44	Q803J2 DANRE Ubiquitin conjugating enzyme E2N OS Danio rerio GN ube2na PE 2 SV 1	142.41	60.2168	121.3046
Q567N5	2	23.28	1.38	Q567N5 DANRE Ribosomal protein L6 OS Danio rerio GN rpl6 PE 2 SV 1	133.48	61.84314	149.535

H9GXR0	3 (2)	20.04	16.25	H9GXR0 DANRE Uncharacterized protein Fragment OS Danio rerio GN zgc 85829 PE 4 SV 1	45.82	12.16716	2.996224
F1RCH5	2 (1)	19.49	1.98	F1RCH5 DANRE Uncharacterized protein OS Danio rerio GN zgc 86599 PE 2 SV 1	31.3	16.21478	16.78735
B0S6H6	4	19.34	1.47	B0S6H6 DANRE Uncharacterized protein OS Danio rerio GN sypb PE 2 SV 1	470.1	356.9558	423.9551
F8W470	3 (1)	19.27	2.81	F8W470 DANRE Uncharacterized protein OS Danio rerio GN sagb PE 4 SV 1	57.33	22.19823	109.0328
Q8AWD9	3	19.24	3.19	Q8AWD9 DANRE Ctsd protein OS Danio rerio GN ctsd PE 2 SV 1	112.96	179.8312	98.76914
B8JJJ5	2	19.19	2.66	B8JJJ5 DANRE Uncharacterized protein OS Danio rerio GN guk1b PE 4 SV 1	303.19	69.04027	262.9452
B8JIS8	3	19.03	1.91	B8JIS8 DANRE Uncharacterized protein OS Danio rerio GN gstm PE 3 SV 1	159.47	50.79864	99.57664
Q6YBS2	2	18.94	1.61	Q6YBS2 DANRE Adenylyl cyclase associated protein OS Danio rerio GN cap1 PE 2 SV 1	182.02	86.67506	120.0083
Q7T1D8	2	18.92	1.57	Q7T1D8 DANRE Phb protein OS Danio rerio GN phb PE 2 SV 1	172.38	66.74641	150.0237
Q7SZC9	2 (1)	18.67	36.56	Q7SZC9 DANRE Heterogeneous nuclear ribonucleoprotein R OS Danio rerio GN hnrnpr PE 2 SV 1	78.27	1.298639	41.58589
Q6NUV2	2 (1)	18.57	2.37	Q6NUV2 DANRE Uncharacterized protein OS Danio rerio GN saga PE 2 SV 1	19	6.141228	25.54228
A7YY99	3 (2)	18.5	1.8	A7YY99 DANRE Uncharacterized protein OS Danio rerio GN atp5ia PE 4 SV 1	134.17	45.18536	79.98431
E9QFY9	3	18.37	8.27	E9QFY9 DANRE Galectin OS Danio rerio GN lgals9l1 PE 4 SV 1	48.71	244.5325	79.87806
Q6PFT7	2	18.32	2.51	Q6PFT7 DANRE Coactosin like 1 OS Danio rerio GN cotl1 PE 2 SV 1	113.17	166.9601	116.3427
B8JK71	3	18.29	1.42	B8JK71 DANRE Uncharacterized protein OS Danio rerio GN cep170a PE 4 SV 1	257.31	119.4809	192.4915
F1QS18	2 (1)	18.12	1.37	F1QS18 DANRE Uncharacterized protein Fragment OS Danio rerio GN LOC100002384 PE 4 SV 1	29.53	13.05921	23.89542
Q803B0	2	17.96	2.53	Q803B0 DANRE Heat shock 60kD protein 1 Chaperonin OS Danio rerio GN hspd1 PE 2 SV 1	96.42	53.13497	40.42777
Q566W6	2	17.88	2.29	Q566W6 DANRE Uncharacterized protein OS Danio rerio GN zgc 112425 PE 2 SV 1	283.19	82.12982	329.2128
Q4V8S2	3	17.74	4.67	Q4V8S2 DANRE Uncharacterized protein OS Danio rerio GN rcvrna PE 2 SV 1	128.64	16.91265	138.3788
F1QDK2	2 (1)	17.69	1.28	F1QDK2 DANRE Uncharacterized protein OS Danio rerio PE 4 SV 1	463.97	248.9989	559.1251
E9QB23	2 (0)	17.68	1	E9QB23 DANRE Uncharacterized protein OS Danio rerio GN si dkey 16p6 1 PE 4 SV 2	0	0	0
Q6DRE6	2	17.66	2.34	Q6DRE6 DANRE 60S ribosomal protein L12 OS Danio rerio GN rpl12 PE 2 SV 1	1703.88	442.2534	989.742
F1QZQ1	2	17.26	1.61	F1QZQ1 DANRE Uncharacterized protein Fragment OS Danio rerio PE 4 SV 1	212.3	119.1896	140.4188
Q6PBW3	2	13.3	1.23	Q6PBW3 DANRE Ribosomal protein S14 OS Danio rerio GN rps14 PE 2 SV 1	88.88	66.31555	96.77166
Q68EH2	2	12.58	6.48	Q68EH2 DANRE Uncharacterized protein OS Danio rerio GN ak1 PE 2 SV 1	101.03	202.4481	54.69703
A5PMV7	2 (1)	12.54	Infinity	A5PMV7 DANRE Uncharacterized protein OS Danio rerio GN ppp2r1a PE 2 SV 1	28.43	12.04579	0
Q6DGL0	2	12.5	1.38	Q6DGL0 DANRE 60S ribosomal protein L18 OS Danio rerio GN rpl18 PE 2 SV 1	75.43	41.36833	100.076
F1QS69	1	12.5	3.19	F1QS69 DANRE Uncharacterized protein OS Danio rerio GN ndufa13 PE 4 SV 1	78.89	15.00717	61.18884
F1RDA6	1	12.46	1.91	F1RDA6 DANRE Uncharacterized protein OS Danio rerio GN ndufs1 PE 4 SV 1	102.32	42.16936	56.93888

F1R8U0	1	12.46	11.65	F1R8U0 DANRE Uncharacterized protein OS Danio rerio GN zgc 153629 PE 3 SV 1	76.3	539.2629	197.687
Q6PC35	1 (0)	12.42	1	Q6PC35 DANRE ATPase H transporting lysosomal V1 subunit H OS Danio rerio GN atp6v1h PE 2 SV 1	0	0	0
F1QYB8	3 (2)	12.41	1.28	F1QYB8 DANRE Uncharacterized protein OS Danio rerio GN rab1bb PE 3 SV 1	291.73	140.4958	241.9291
F1Q8I5	1	12.38	1.12	F1Q8I5 DANRE Uncharacterized protein OS Danio rerio GN lsm14aa PE 2 SV 1	956.77	609.4077	953.7661
Q285P3	1	12.3	3.69	Q285P3 DANRE Facilitated glucose transporter 1 OS Danio rerio GN slc2a1a PE 2 SV 1	237.34	346.8762	164.6436
Q6NYJ9	2	12.26	3.33	Q6NYJ9 DANRE Adaptor related protein complex 2 beta 1 subunit OS Danio rerio GN ap2b1 PE 2 SV 1	187.96	34.20761	77.35995
B0UYN4	2	12.25	1.22	B0UYN4 DANRE Uncharacterized protein OS Danio rerio GN zgc 162944 PE 4 SV 1	55.85	40.94354	72.52562
F6NL35	2	12.25	4.1	F6NL35 DANRE Uncharacterized protein OS Danio rerio GN si ch211 214j24 9 PE 2 SV 1	161.05	23.83063	122.2289
Q6PFS4	2	12.25	12.09	Q6PFS4 DANRE Inner membrane protein mitochondrial Mitofilin OS Danio rerio GN immt PE 2 SV 1	91.81	7.998161	8.07493
Q6PBJ8	3	12.25	1.4	Q6PBJ8 DANRE Peptidyl prolyl cis trans isomerase OS Danio rerio GN fkbp1aa PE 4 SV 1	142.68	61.79459	138.6657
Q568W2	1	12.24	2.38	Q568W2 DANRE Atp6v1b2 protein OS Danio rerio GN atp6v1b2 PE 2 SV 1	183.25	85.8983	81.80116
B0S782	1 (0)	12.1	1	B0S782 DANRE Uncharacterized protein OS Danio rerio GN si dkey 199k11 6 PE 4 SV 1	0	0	0
E9QFG9	1	12.1	8.92	E9QFG9 DANRE Uncharacterized protein OS Danio rerio GN si dkey 22i16 2 PE 2 SV 1	272.93	108.4849	32.50159
Q803J3	2	11.98	2.12	Q803J3 DANRE RAB2A member RAS oncogene family OS Danio rerio GN rab2a PE 2 SV 1	145.14	41.58073	95.43292
Q804G7	1	11.96	7.23	Q804G7 DANRE Annexin OS Danio rerio GN anxa4 PE 2 SV 1	10.83	47.50956	29.49474
Q6PBW8	1	11.82	1.47	Q6PBW8 DANRE Uncharacterized protein OS Danio rerio GN lypla2 PE 2 SV 1	103.27	42.69124	75.14997
Q6PBR5	1	11.82	1.19	Q6PBR5 DANRE ATPase H transporting V1 subunit G isoform 1 OS Danio rerio GN atp6v1g1 PE 4 SV 1	170.96	107.7992	158.9805
E7F8T4	1	11.76	1.9	E7F8T4 DANRE Uncharacterized protein OS Danio rerio GN LOC100006782 PE 4 SV 1	226.55	173.1741	159.7242
F1R3A7	2 (1)	11.67	2.37	F1R3A7 DANRE Uncharacterized protein OS Danio rerio GN si dkey 190j3 4 PE 4 SV 2	27.92	7.154652	13.39801
F1Q912	2 (1)	11.63	19.63	F1Q912 DANRE Uncharacterized protein OS Danio rerio GN si dkey 261p22 1 PE 4 SV 4	6.59	52.43711	4.674959
E7F2M5	1	11.6	8.89	E7F2M5 DANRE Uncharacterized protein OS Danio rerio GN BX957297 2 PE 4 SV 1	260.47	261.8821	51.56268
F1RE68	1	11.54	1.85	F1RE68 DANRE Uncharacterized protein OS Danio rerio GN srsf11 PE 2 SV 1	78.57	62.20117	58.74511
Q1LYH0	1	11.52	1.93	Q1LYH0 DANRE Uncharacterized protein Fragment OS Danio rerio GN si dkey 110c1 7 PE 4 SV 1	118.68	42.15116	65.26881
E9QDX1	2 (1)	11.51	3.54	E9QDX1 DANRE Uncharacterized protein OS Danio rerio GN si dkey 222h21 6 PE 4 SV 1	28.11	4.824384	15.06612
Q6DH51	1 (0)	11.46	1	Q6DH51 DANRE Polyglutamine binding protein 1 like OS Danio rerio GN pqbp1 PE 2 SV 1	0	0	0
F1QHM2	1	11.2	3.17	F1QHM2 DANRE Uncharacterized protein Fragment OS Danio rerio GN LOC556118 PE 3 SV 1	77.77	14.87367	49.76707
I3ITE4	1	6.71	8.03	I3ITE4 DANRE Acidic leucine rich nuclear phosphoprotein 32 family member E Fragment OS Danio rerio	107.96	8.155939	44.05087
B0S5B9	1	6.42	2.74	B0S5B9 DANRE Uncharacterized protein OS Danio rerio GN sypa PE 4 SV 1	181.25	139.9799	89.29172
Q6DGZ5	1	6.41	4.36	Q6DGZ5 DANRE Guanine nucleotide binding protein subunit gamma OS Danio rerio GN gng7 PE 3 SV 1	19.21	6.432512	4.685584

Q5XJS8	1	6.39	2.44	Q5XJS8 DANRE Hydroxysteroid 17 beta dehydrogenase 10 OS Danio rerio GN hsd17b10 PE 2 SV 1	76.55	23.05388	33.27721
Q567H2	1	6.31	3.63	Q567H2 DANRE Uncharacterized protein OS Danio rerio GN zgc 112056 PE 2 SV 1	55.1	17.56804	16.12861
B0S5R8	1	6.31	74.05	B0S5R8 DANRE Uncharacterized protein OS Danio rerio GN LOC100535341 PE 4 SV 1	55.85	8.49577	0.796868
F1R9S7	1	6.27	12.37	F1R9S7 DANRE Uncharacterized protein Fragment OS Danio rerio GN pdca PE 4 SV 1	56.61	2.77933	46.18647
Q6P937	1	6.25	2.28	Q6P937 DANRE Clathrin light chain Lca OS Danio rerio GN clta PE 2 SV 1	77.02	37.49669	35.96531
F1QJV7	1	6.25	2.61	F1QJV7 DANRE Uncharacterized protein OS Danio rerio GN ddt PE 4 SV 1	49.16	11.41467	25.05353
B8JIQ1	1	6.2	3.03	B8JIQ1 DANRE Uncharacterized protein OS Danio rerio GN si dkeyp 80c12 7 PE 4 SV 1	140.02	216.806	450.7086
Q5RI07	1	6.19	13.64	Q5RI07 DANRE Uncharacterized protein OS Danio rerio GN pdcb PE 2 SV 1	69.67	3.100956	35.94406
H9GYP7	1	6.17	22.14	H9GYP7 DANRE Plastin 2 Fragment OS Danio rerio GN lcp1 PE 4 SV 1	1.82	24.45568	2.762476
E7F6Q2	1	6.16	1.29	E7F6Q2 DANRE Dihydropyrimidinase related protein 3 OS Danio rerio GN dpysl3 PE 2 SV 1	99.22	59.99227	81.74804
Q6NWH2	1	6.13	2.2	Q6NWH2 DANRE MARCKS like 2 OS Danio rerio GN marcksl1b PE 2 SV 1	17.34	20.64472	16.43673
Q6P3K5	2	6.1	3.25	Q6P3K5 DANRE Krt5 protein OS Danio rerio GN krt5 PE 2 SV 1	75.53	149.0583	152.1274
Q90Z34	1	6.1	4.35	Q90Z34 DANRE ATPase Na K transporting beta 2b polypeptide OS Danio rerio GN atp1b2b PE 2 SV 1	55.57	7.755424	20.82482
Q6P948	1	6.08	2.21	Q6P948 DANRE Pdha1 protein OS Danio rerio GN pdha1a PE 2 SV 1	33.36	14.27289	16.02236
Q6DRC0	1	6.05	2.09	Q6DRC0 DANRE Seryl tRNA synthetase OS Danio rerio GN sars PE 2 SV 1	60.12	25.99706	30.62098
F1R541	2 (1)	6.05	2.47	F1R541 DANRE Uncharacterized protein Fragment OS Danio rerio GN rab1ba PE 2 SV 1	36.36	9.594152	41.46901
B3DIV6	1	6.04	5.03	B3DIV6 DANRE Dihydrolipoamide S acetyltransferase E2 component of pyruvate dehydrogenase complex O	47.66	5.75285	27.87976
G1K2H6	1	6.03	1.46	G1K2H6 DANRE Keratin type I cytoskeletal 18 OS Danio rerio GN krt18 PE 2 SV 1	44.99	39.97867	62.62321
Q7ZUS9	1	6.03	3.69	Q7ZUS9 DANRE Electron transfer flavoprotein alpha polypeptide OS Danio rerio GN etfa PE 2 SV 1	43.96	10.38304	12.65427
F1QFC0	1	6.03	2.22	F1QFC0 DANRE Uncharacterized protein OS Danio rerio GN hspa9 PE 3 SV 1	21.71	10.05535	39.04654
F1QPX6	1	5.98	4.61	F1QPX6 DANRE Uncharacterized protein Fragment OS Danio rerio GN zgc 152830 PE 4 SV 1	24.74	33.9406	12.88801
Q5TZ35	1	5.95	3.88	Q5TZ35 DANRE Si dkey 30j22 11 OS Danio rerio GN vsnl1a PE 2 SV 1	54.79	8.562523	31.29035
F1QS28	1	5.93	5.31	F1QS28 DANRE Uncharacterized protein OS Danio rerio GN hnrnpa0a PE 4 SV 1	116.65	13.34443	70.84688
Q6DH14	2	5.93	3.42	Q6DH14 DANRE Beta A1 crystallin OS Danio rerio GN cryba1a PE 2 SV 1	120.03	209.9062	107.5984
B8JIS3	1	5.84	2.26	B8JIS3 DANRE Uncharacterized protein OS Danio rerio GN eps8l3 PE 2 SV 1	72.26	19.38249	66.92629
E9QC84	1	5.83	38.91	E9QC84 DANRE Uncharacterized protein OS Danio rerio GN ap2b1 PE 4 SV 1	27.61	0.430857	18.97609
Q7ZUI4	1	5.82	1.31	Q7ZUI4 DANRE Thioredoxin OS Danio rerio GN zgc 56493 PE 3 SV 1	42.49	24.6256	34.48845
F1R096	1	5.81	Infinity	F1R096 DANRE Uncharacterized protein OS Danio rerio GN sh3bp5lb PE 4 SV 2	0	138.5417	0

E7FDL7	1	5.79	6.42	E7FDL7 DANRE Uncharacterized protein OS Danio rerio GN LOC100331323 PE 4 SV 1	53.99	5.10353	22.39731
Q6P5M3	1	5.68	1.32	Q6P5M3 DANRE Ribosomal protein S9 OS Danio rerio GN rps9 PE 2 SV 1	29.4	16.9612	39.18466
F1R443	1	5.67	10.98	F1R443 DANRE Uncharacterized protein OS Danio rerio GN spna2 PE 2 SV 1	23.77	1.310776	13.39801
Q24JW2	1	5.63	Infinity	Q24JW2 DANRE Lysozyme OS Danio rerio GN lyz PE 2 SV 1	0	66.16991	0
E9QDC7	1	0	2.22	E9QDC7 DANRE Uncharacterized protein OS Danio rerio GN si dkey 211i20 2 PE 4 SV 3	50.03	40.48234	117.8621
Q6IQ59	1	0	2.04	Q6IQ59 DANRE Ubiquinol cytochrome c reductase core protein II OS Danio rerio GN uqcrc2b PE 2 SV 1			

Comparison of proteomic profiles in the zebrafish retina during experimental degeneration and regeneration.

Authors: **Karen Eastlake, Wendy E. Heywood, Dhani Tracey-White, Erika Aquino, Emily Bliss, Gerardo R Vasta, Kevin Mills, Peng T Khaw, Mariya Moosajee , G. Astrid Limb***

Supplementary Table 2: Gene ontology enrichment of proteins differentially expressed between degenerated and normal retina as conducted by Panther online database

GO annotations, with P-values and FDR of proteins differentially expressed between control and degenerated retina as identified by Webgestalt and Panther online databases.

Analysis Type:	PANTHER Overrepresentation Test (release 20160715)					
Annotation Version and Release Date:	GO Ontology database Released 2016-07-29					
Analyzed List:	upload_1 (Danio rerio)					
Reference List:	Danio rerio (all genes in database)					
Bonferroni correction:	TRUE					
GO cellular component complete	Danio rerio - REFLIST (27187)	upload_1 (141)	upload_1 (expected)	upload_1 (over/under)	upload_1 (fold Enrichment)	upload_1 (P-value)
fibrinogen complex (GO:0005577)	3	3	0.02	+	> 100	3.95E-04
proton-transporting ATP synthase complex, catalytic core F(1) (GO:0045261)	5	3	0.03	+	> 100	1.81E-03
mitochondrial proton-transporting ATP synthase complex, coupling factor F(o) (GO:0000276)	9	4	0.05	+	85.7	1.19E-04
proton-transporting ATP synthase complex (GO:0045259)	19	7	0.1	+	71.04	9.21E-09
mitochondrial proton-transporting ATP synthase complex (GO:0005753)	12	4	0.06	+	64.27	3.71E-04
proton-transporting ATP synthase complex, coupling factor F(o) (GO:0045263)	13	4	0.07	+	59.33	5.09E-04
proton-transporting two-sector ATPase complex, catalytic domain (GO:0033178)	19	4	0.1	+	40.59	2.27E-03
proton-transporting two-sector ATPase complex, proton-transporting domain (GO:0033177)	25	5	0.13	+	38.56	1.66E-04
proton-transporting two-sector ATPase complex (GO:0016469)	46	9	0.24	+	37.72	2.83E-09
nucleosome (GO:0000786)	51	5	0.26	+	18.9	5.28E-03
inner mitochondrial membrane protein complex (GO:0098800)	62	6	0.32	+	18.66	6.88E-04
DNA packaging complex (GO:0044815)	55	5	0.29	+	17.53	7.57E-03
intermediate filament (GO:0005882)	56	5	0.29	+	17.22	8.25E-03

intermediate filament cytoskeleton (GO:0045111)	57	5	0.3	+	16.91	8.98E-03
mitochondrial membrane part (GO:0044455)	92	7	0.48	+	14.67	4.20E-04
mitochondrial protein complex (GO:0098798)	79	6	0.41	+	14.64	2.74E-03
protein-DNA complex (GO:0032993)	71	5	0.37	+	13.58	2.54E-02
organelle inner membrane (GO:0019866)	205	12	1.06	+	11.29	7.12E-07
mitochondrial inner membrane (GO:0005743)	178	10	0.92	+	10.83	2.66E-05
mitochondrial membrane (GO:0031966)	264	13	1.37	+	9.49	1.10E-06
polymeric cytoskeletal fiber (GO:0099513)	208	10	1.08	+	9.27	1.11E-04
supramolecular fiber (GO:0099512)	208	10	1.08	+	9.27	1.11E-04
mitochondrial envelope (GO:0005740)	284	13	1.47	+	8.83	2.61E-06
organelle envelope (GO:0031967)	405	15	2.1	+	7.14	2.69E-06
envelope (GO:0031975)	407	15	2.11	+	7.11	2.87E-06
mitochondrial part (GO:0044429)	377	13	1.96	+	6.65	6.91E-05
membrane protein complex (GO:0098796)	631	16	3.27	+	4.89	1.43E-04
mitochondrion (GO:0005739)	634	16	3.29	+	4.87	1.52E-04
cytoskeletal part (GO:0044430)	528	12	2.74	+	4.38	1.47E-02
organelle membrane (GO:0031090)	779	17	4.04	+	4.21	4.63E-04
intracellular organelle part (GO:0044446)	2674	37	13.87	+	2.67	1.41E-05
organelle part (GO:0044422)	2721	37	14.11	+	2.62	2.21E-05
protein complex (GO:0043234)	2243	27	11.63	+	2.32	2.10E-02
cytoplasmic part (GO:0044444)	2753	33	14.28	+	2.31	2.46E-03
macromolecular complex (GO:0032991)	2662	31	13.81	+	2.25	9.25E-03
cytoplasm (GO:0005737)	4504	46	23.36	+	1.97	1.47E-03
intracellular (GO:0005622)	7688	69	39.87	+	1.73	1.12E-04
intracellular part (GO:0044424)	7368	65	38.21	+	1.7	7.03E-04
cell (GO:0005623)	9420	72	48.85	+	1.47	2.97E-02
cell part (GO:0044464)	9420	72	48.85	+	1.47	2.97E-02
Unclassified (UNCLASSIFIED)	12225	43	63.4	-	0.68	0.00E+00

Comparison of proteomic profiles in the zebrafish retina during experimental degeneration and regeneration

Authors: **Karen Eastlake, Wendy E. Heywood, Dhani Tracey-White, Erika Aquino, Emily Bliss, Gerardo R Vasta, Kevin Mills, Peng T Khaw, Mariya Moosajee , G. Astrid Limb***

Supplementary Table 3: Gene ontology enrichment of proteins differentially expressed between regenerating and normal retina as conducted by Panther online database

GO annotations, with P-values and FDR of proteins differentially expressed between control and regenerating retina as identified by Webgestalt and Panther online databases.

Analysis Type:	PANTHER Overrepresentation Test (release 20160715)					
Annotation Version and Release Date:	GO Ontology database Released 2016-07-29					
Analyzed List:	upload_1 (Danio rerio)					
Reference List:	Danio rerio (all genes in database)					
Bonferroni correction:	TRUE					
GO cellular component complete	Danio rerio - REFLIST (27187)	upload_1 (40)	upload_1 (expected)	upload_1 (over/under)	upload_1 (fold Enrichment)	upload_1 (P-value)
microtubule (GO:0005874)	136	4	0.2	+	19.99	3.22E-02
mitochondrial inner membrane (GO:0005743)	178	5	0.26	+	19.09	4.25E-03
mitochondrial membrane (GO:0031966)	264	7	0.39	+	18.02	7.45E-05
mitochondrial envelope (GO:0005740)	284	7	0.42	+	16.75	1.22E-04
organelle inner membrane (GO:0019866)	205	5	0.3	+	16.58	8.37E-03
polymeric cytoskeletal fiber (GO:0099513)	208	5	0.31	+	16.34	8.97E-03
supramolecular fiber (GO:0099512)	208	5	0.31	+	16.34	8.97E-03
mitochondrial part (GO:0044429)	377	8	0.55	+	14.42	4.60E-05
organelle envelope (GO:0031967)	405	7	0.6	+	11.75	1.28E-03
envelope (GO:0031975)	407	7	0.6	+	11.69	1.32E-03
mitochondrion (GO:0005739)	634	8	0.93	+	8.58	2.24E-03
organelle membrane (GO:0031090)	779	8	1.15	+	6.98	9.99E-03
intracellular organelle part (GO:0044446)	2674	15	3.93	+	3.81	1.84E-03
organelle part (GO:0044422)	2721	15	4	+	3.75	2.29E-03
cytoplasm (GO:0005737)	4504	18	6.63	+	2.72	1.56E-02
intracellular part (GO:0044424)	7368	23	10.84	+	2.12	3.27E-02
Unclassified (UNCLASSIFIED)	12225	11	17.99	-	0.61	0.00E+00
